# Supplementary material for: COVID-19 Vaccine Uptake and Effectiveness by Time since Vaccination in the Western Cape Province, South Africa: An Observational Cohort Study during 2020–2022
Source: Vaccines (Basel). 2024 Jun 5;12(6):628. doi: 10.3390/vaccines12060628 (PMC11209070; doi:10.3390/vaccines12060628)

Supplementary Material 2. Analysis Outputs

This document provides supplementary analysis outputs for the following article:

COVID-19 vaccine uptake and effectiveness by time since vaccination in the Western Cape province, South Africa: An observational cohort study during 2020-2022

Reshma Kassanjee, Mary-Ann Davies, Alexa Heekes, Hassan Mahomed, Anthony J Hawkridge, Erna Morden, Theuns Jacobs, Cheryl Cohen, Harry Moultrie, Richard J Lessells, Nicolette Van Der Walt, Juanita O Arendse, Nicole Wolter, Sibongile Walaza, Waasila Jassat, Anne von Gottberg, Patrick L Hannan, Daniel Feikin, Keith Cloete, Andrew Boulle

2024

Table of contents

S2.1. Description of cohort **3**

S2.1.1 Cohort description by vaccination status 3

S2.1.2 Cohort description by age group 6

S2.2. COVID-19 outcomes, SARS-CoV-2 testing and positivity, and COVID-19 vaccination over time **9**

S2.2.1 Outcomes, testing and vaccination over time: Males 10

S2.2.2 Outcomes, testing and vaccination over time: Females 11

S2.2.3 Outcomes, testing and vaccination over time: Age 18-34 years 12

S2.2.4 Outcomes, testing and vaccination over time: Age 35-49 years 13

S2.2.5 Outcomes, testing and vaccination over time: Age 50-59 years 14

S2.2.6 Outcomes, testing and vaccination over time: Age 60+ years 15

S2.2.7 Outcomes, testing and vaccination over time: Living with HIV at start 2020 16

S2.2.8 Outcomes, testing and vaccination over time: Not known to be living with HIV at start 2020 17

S2.3. Vaccine effectiveness for distinct subpopulations **18**

S2.3.1 Vaccine effectiveness: Males 18

S2.3.2 Vaccine effectiveness: Females 19

S2.3.3 Vaccine effectiveness: Age 18-49 years 20

S2.3.4 Vaccine effectiveness: Age 50+ years 21

S2.3.5 Vaccine effectiveness: Known to be living with HIV at start 2020 22

S2.3.6 Vaccine effectiveness: Not known to be living with HIV at start 2020 23

S2.3.7 Vaccine effectiveness: Excluding people who received heterologous vaccines 24

S2.4. All multivariable cox model hazard ratios **25**

S2.4.1 Hazard ratios: Demographic characteristics 25

S2.4.2 Hazard ratios: Non-communicable conditions 26

S2.4.3 Hazard ratios: Infectious conditions 26

S2.4.4 Hazard ratios: Other conditions 27

S2.4.5 Hazard ratios: Prior SARS-CoV-2 diagnosis 27

S2.4.6 Hazard ratios: Location 28

S2.4.7 Hazard ratios: Healthcare utilisation and testing 29

S2.4.8 Hazard ratios: Vaccine terms 30

S2.5. Vaccine effectiveness for rolling windows of calendar time **31**

S2.1. Description of cohort

The tables below report on all variables considered in this study. The cohort, which is followed during calendar years 2020 to 2022, is stratified by completion of a primary COVID-19 vaccination series by end 2022, or age group at start 2020. A subset of these variables is also reported in Tables 1 and 2 of the article. Percent (and count frequency) are reported.

S2.1.1 Cohort description by vaccination status

		**Complete vaccination[a] by end 2022**	
	**Overall**<br>N = 2,429,927	**No**<br>N = 1,455,385	**Yes**<br>N = 974,542
**Age category at start 2020 (years)**			
18-34	41.6 (1,009,672)	46.6 (677,813)	34.1 (331,859)
35-49	29.3 (711,077)	27.2 (395,497)	32.4 (315,580)
50-59	14.1 (342,308)	12.4 (179,961)	16.7 (162,347)
60-99	15.1 (366,870)	13.9 (202,114)	16.9 (164,756)
**Sex**			
Female	59.7 (1,450,660)	57.8 (841,578)	62.5 (609,082)
Male	40.3 (979,267)	42.2 (613,807)	37.5 (365,460)
**PHC visit location type**			
Cape Town Metro	51.4 (1,248,277)	51.0 (742,774)	51.9 (505,503)
Other (non-Metro or no visit recorded)	48.6 (1,181,650)	49.0 (712,611)	48.1 (469,039)
**Location (subdistrict or district)**			
S01	7.3 (176,386)	7.4 (107,731)	7.0 (68,655)
S02	3.9 (94,234)	3.9 (57,051)	3.8 (37,183)
S03	6.1 (147,607)	5.6 (81,768)	6.8 (65,839)
S04	9.4 (227,820)	9.1 (132,798)	9.8 (95,022)
S05	13.4 (325,336)	12.6 (183,230)	14.6 (142,106)
S06	1.4 (35,067)	1.6 (23,180)	1.2 (11,887)
S07	11.4 (276,582)	12.5 (181,870)	9.7 (94,712)
S08	8.3 (200,836)	8.3 (121,121)	8.2 (79,715)
S09	6.3 (153,580)	6.4 (92,565)	6.3 (61,015)
S10	8.9 (215,155)	9.0 (130,917)	8.6 (84,238)
S11	5.3 (127,820)	5.1 (73,827)	5.5 (53,993)
S12	11.3 (273,812)	11.2 (162,500)	11.4 (111,312)
S13	7.2 (175,692)	7.3 (106,827)	7.1 (68,865)

	Overall<br>N = 2,429,927	Complete vaccination[a] by end 2022<br>No<br>N = 1,455,385	 Yes<br>N = 974,542
**Number of PHC facility visits (during 5 years before the pandemic)**			
0	17.2 (418,046)	17.8 (258,816)	16.3 (159,230)
1-4	31.3 (759,411)	33.8 (492,631)	27.4 (266,780)
5-14	23.4 (567,415)	23.7 (345,062)	22.8 (222,353)
15+	28.2 (685,055)	24.7 (358,876)	33.5 (326,179)
**Number of years with PHC facility visits (out of the 3 years before the pandemic)**			
0	26.9 (654,432)	28.9 (420,879)	24.0 (233,553)
1	24.5 (594,713)	26.4 (384,147)	21.6 (210,566)
2	18.1 (440,456)	18.6 (270,732)	17.4 (169,724)
3	30.5 (740,326)	26.1 (379,627)	37.0 (360,699)
**Hypertension**			
Absent by end 2022	76.5 (1,858,079)	79.8 (1,161,884)	71.4 (696,195)
Onset during 2020-2022	3.4 (83,174)	2.8 (40,473)	4.4 (42,701)
Present before 2020	20.1 (488,674)	17.4 (253,028)	24.2 (235,646)
**Diabetes**			
Absent by end 2022	89.5 (2,174,691)	90.8 (1,321,701)	87.5 (852,990)
Onset during 2020-2022	1.9 (46,111)	1.6 (22,634)	2.4 (23,477)
Present before 2020	8.6 (209,125)	7.6 (111,050)	10.1 (98,075)
**Chronic kidney disease**			
Absent by end 2022	95.7 (2,324,768)	95.9 (1,396,419)	95.3 (928,349)
Onset during 2020-2022	0.7 (16,445)	0.5 (7,707)	0.9 (8,738)
Present before 2020	3.7 (88,714)	3.5 (51,259)	3.8 (37,455)
**Chronic respiratory disease**			
Absent by end 2022	92.2 (2,241,094)	92.5 (1,346,451)	91.8 (894,643)
Onset during 2020-2022	1.5 (36,451)	1.4 (20,454)	1.6 (15,997)
Present before 2020	6.3 (152,382)	6.1 (88,480)	6.6 (63,902)
**HIV**			
Absent by end 2022	85.4 (2,075,796)	85.3 (1,241,925)	85.6 (833,871)
Onset during 2020-2022	1.4 (32,986)	1.4 (21,048)	1.2 (11,938)
Present before 2020	13.2 (321,145)	13.2 (192,412)	13.2 (128,733)
**Tuberculosis (at any time in past)[b]**			
Absent by end 2022	90.2 (2,190,920)	89.2 (1,298,552)	91.6 (892,368)
(First) onset during 2020-2022	1.7 (42,068)	2.0 (28,783)	1.4 (13,285)
Present before 2020	8.1 (196,939)	8.8 (128,050)	7.1 (68,889)
**Tuberculosis (ongoing episode)**			
Absent throughout 2020-2022	96.8 (2,351,220)	96.2 (1,400,628)	97.5 (950,592)
Experienced episode during 2020-2022	3.2 (78,707)	3.8 (54,757)	2.5 (23,950)
**Pregnancy (ongoing episode)**			
Absent throughout 2020-2022	91.5 (2,222,462)	90.6 (1,319,172)	92.7 (903,290)
Experienced during 2020-2022	8.5 (207,465)	9.4 (136,213)	7.3 (71,252)

	Overall<br>N = 2,429,927	Complete vaccination[a] by end 2022<br>No<br>N = 1,455,385	Yes<br>N = 974,542
**Number of negative SARS-CoV-2 tests (by end 2022)**			
0	81.8 (1,987,267)	84.3 (1,227,129)	78.0 (760,138)
1	13.1 (317,758)	11.4 (165,723)	15.6 (152,035)
2+	5.1 (124,902)	4.3 (62,533)	6.4 (62,369)
**COVID-19 vaccination status[a] and boosters (at end 2022)**			
None	55.0 (1,336,826)	91.9 (1,336,826)	0.0 (0)
Incomplete BNT162b2	4.9 (118,559)	8.1 (118,559)	0.0 (0)
Complete Ad26.COV2S (no booster)	9.7 (236,725)	0.0 (0)	24.3 (236,725)
Complete Ad26.COV2S (and booster)	1.7 (41,196)	0.0 (0)	4.2 (41,196)
Complete BNT162b2 (no booster)	22.6 (548,914)	0.0 (0)	56.3 (548,914)
Complete BNT162b2 (and booster)	6.1 (147,707)	0.0 (0)	15.2 (147,707)
**Vital status (at end 2022)**			
Alive	95.4 (2,318,859)	93.3 (1,357,339)	98.7 (961,520)
Died: not COVID-19 related	4.1 (99,525)	6.0 (87,001)	1.3 (12,524)
Died: COVID-19 related	0.5 (11,543)	0.8 (11,045)	0.1 (498)
**SARS-CoV-2 diagnosis (by end 2022)**			
Not experienced	92.1 (2,237,396)	93.3 (1,357,422)	90.3 (879,974)
Experienced (2020-2022)	7.9 (192,531)	6.7 (97,963)	9.7 (94,568)
**COVID-19 related hospitalisation or death (by end 2022)**			
Not experienced	98.4 (2,391,586)	98.2 (1,429,692)	98.7 (961,894)
Experienced (2020-2022)	1.6 (38,341)	1.8 (25,693)	1.3 (12,648)
**COVID-19 related hospitalisation with severe disease or death (by end 2022)**			
Not experienced	99.3 (2,412,279)	99.0 (1,441,138)	99.7 (971,141)
Experienced (2020-2022)	0.7 (17,648)	1.0 (14,247)	0.3 (3,401)
**COVID-19 related death (by end 2022)**			
Not experienced	99.5 (2,418,384)	99.2 (1,444,340)	99.9 (974,044)
Experienced (2020-2022)	0.5 (11,543)	0.8 (11,045)	0.1 (498)

PHC: Primary healthcare
[a] Complete vaccination: ≥28 days since a first Ad26.COV2.S dose or ≥14 days since a second dose following a first BNT162b2 dose; incomplete BNT162b2: ≥21 days since a first BNT162b2 dose but not yet completely vaccinated (see methods).
[b] Tuberculosis (at any time in past) includes those with an ongoing episode.

S2.1.2 Cohort description by age group

Characteristic	Overall	Age category (years) at start 2020			
		18-34	35-49	50-59	60-99
	N = 2,429,927	N = 1,009,672	N = 711,077	N = 342,308	N = 366,870
**Sex**					
Female	59.7 (1,450,660)	62.0 (626,011)	57.6 (409,758)	56.3 (192,795)	60.5 (222,096)
Male	40.3 (979,267)	38.0 (383,661)	42.4 (301,319)	43.7 (149,513)	39.5 (144,774)
**PHC visit location type**					
Cape Town Metro	51.4 (1,248,277)	52.5 (530,567)	51.2 (363,887)	48.8 (167,199)	50.9 (186,624)
Other (non-Metro or no visit recorded)	48.6 (1,181,650)	47.5 (479,105)	48.8 (347,190)	51.2 (175,109)	49.1 (180,246)
**Location (subdistrict or district)**					
S01	7.3 (176,386)	7.7 (77,751)	7.4 (52,369)	6.8 (23,243)	6.3 (23,023)
S02	3.9 (94,234)	4.2 (42,196)	4.0 (28,349)	3.2 (11,123)	3.4 (12,566)
S03	6.1 (147,607)	5.2 (52,567)	5.7 (40,740)	6.7 (22,812)	8.6 (31,488)
S04	9.4 (227,820)	8.7 (87,823)	9.5 (67,544)	9.6 (32,846)	10.8 (39,607)
S05	13.4 (325,336)	13.5 (136,218)	13.3 (94,447)	14.2 (48,470)	12.6 (46,201)
S06	1.4 (35,067)	1.3 (13,396)	1.5 (10,403)	1.6 (5,604)	1.5 (5,664)
S07	11.4 (276,582)	11.0 (110,967)	11.5 (81,646)	12.1 (41,279)	11.6 (42,690)
S08	8.3 (200,836)	9.8 (99,447)	8.7 (62,133)	6.3 (21,431)	4.9 (17,825)
S09	6.3 (153,580)	6.2 (62,751)	6.2 (43,887)	6.3 (21,576)	6.9 (25,366)
S10	8.9 (215,155)	9.5 (96,164)	9.0 (63,647)	7.6 (26,156)	8.0 (29,188)
S11	5.3 (127,820)	5.4 (54,433)	5.2 (37,277)	5.1 (17,404)	5.1 (18,706)
S12	11.3 (273,812)	10.1 (102,187)	11.0 (78,196)	12.9 (44,020)	13.5 (49,409)
S13	7.2 (175,692)	7.3 (73,772)	7.1 (50,439)	7.7 (26,344)	6.9 (25,137)

Characteristic	Overall	Age category (years) at start 2020			
		18-34	35-49	50-59	60-99
	N = 2,429,927	N = 1,009,672	N = 711,077	N = 342,308	N = 366,870
**Number of PHC facility visits (during 5 years before the pandemic)**					
0	17.2 (418,046)	16.5 (166,823)	17.8 (126,341)	17.5 (59,992)	17.7 (64,890)
1-4	31.3 (759,411)	37.7 (380,587)	31.4 (223,158)	24.8 (84,912)	19.3 (70,754)
5-14	23.4 (567,415)	27.4 (276,607)	21.7 (154,654)	19.9 (68,030)	18.6 (68,124)
15+	28.2 (685,055)	18.4 (185,655)	29.1 (206,924)	37.8 (129,374)	44.5 (163,102)
**Number of years with PHC facility visits (out of the 3 years before the pandemic)**					
0	26.9 (654,432)	26.4 (266,477)	28.7 (203,854)	26.3 (90,179)	25.6 (93,922)
1	24.5 (594,713)	29.1 (294,248)	24.1 (171,661)	19.9 (68,264)	16.5 (60,540)
2	18.1 (440,456)	22.0 (221,637)	17.1 (121,763)	14.9 (50,967)	12.6 (46,089)
3	30.5 (740,326)	22.5 (227,310)	30.1 (213,799)	38.8 (132,898)	45.3 (166,319)
**Hypertension**					
Absent at end 2022	76.5 (1,858,079)	94.8 (957,219)	79.2 (563,427)	55.5 (190,033)	40.2 (147,400)
Onset during 2020-2022	3.4 (83,174)	1.7 (17,495)	4.4 (31,142)	5.6 (19,083)	4.2 (15,454)
Present before 2020	20.1 (488,674)	3.5 (34,958)	16.4 (116,508)	38.9 (133,192)	55.6 (204,016)
**Diabetes**					
Absent at end 2022	89.5 (2,174,691)	97.8 (987,299)	91.8 (652,528)	80.3 (274,806)	70.9 (260,058)
Onset during 2020-2022	1.9 (46,111)	0.7 (7,051)	1.9 (13,643)	3.6 (12,180)	3.6 (13,237)
Present before 2020	8.6 (209,125)	1.5 (15,322)	6.3 (44,906)	16.2 (55,322)	25.5 (93,575)
**Chronic kidney disease**					
Absent at end 2022	95.7 (2,324,768)	99.6 (1,005,786)	98.1 (697,237)	94.6 (323,845)	81.2 (297,900)
Onset during 2020-2022	0.7 (16,445)	0.1 (1,061)	0.5 (3,576)	1.1 (3,866)	2.2 (7,942)
Present before 2020	3.7 (88,714)	0.3 (2,825)	1.4 (10,264)	4.3 (14,597)	16.6 (61,028)
**Chronic respiratory disease**					
Absent at end 2022	92.2 (2,241,094)	97.1 (980,010)	93.7 (665,959)	86.2 (294,983)	81.8 (300,142)
Onset during 2020-2022	1.5 (36,451)	0.7 (7,179)	1.4 (10,079)	2.5 (8,717)	2.9 (10,476)
Present before 2020	6.3 (152,382)	2.2 (22,483)	4.9 (35,039)	11.3 (38,608)	15.3 (56,252)
**HIV**					
Absent at end 2022	85.4 (2,075,796)	85.2 (860,276)	77.9 (553,891)	89.4 (305,946)	97.0 (355,683)
Onset during 2020-2022	1.4 (32,986)	2.1 (21,170)	1.3 (8,973)	0.6 (2,127)	0.2 (716)
Present before 2020	13.2 (321,145)	12.7 (128,226)	20.8 (148,213)	10.0 (34,235)	2.9 (10,471)
**Tuberculosis (at any time in past)[a]**					
Absent at end 2022	90.2 (2,190,920)	91.5 (923,917)	86.9 (617,997)	88.8 (303,895)	94.1 (345,111)
(First) onset during 2020-2022	1.7 (42,068)	1.9 (19,093)	1.9 (13,683)	1.6 (5,604)	1.0 (3,688)
Present before 2020	8.1 (196,939)	6.6 (66,662)	11.2 (79,397)	9.6 (32,809)	4.9 (18,071)
**Tuberculosis (ongoing episode)**					
Absent throughout 2020-2022	96.8 (2,351,220)	96.7 (976,653)	96.0 (682,764)	96.8 (331,198)	98.3 (360,605)
Experienced an episode during 2020-2022	3.2 (78,707)	3.3 (33,019)	4.0 (28,313)	3.2 (11,110)	1.7 (6,265)
**Pregnancy (ongoing episode)**					
Absent throughout 2020-2022	91.5 (2,222,462)	82.4 (832,093)	95.8 (681,306)	100.0 (342,193)	100.0 (366,870)
Experienced during 2020-2022	8.5 (207,465)	17.6 (177,579)	4.2 (29,771)	0.0 (115)	0.0 (0)

Characteristic	Overall<br>N = 2,429,927	Age category (years) at start 2020<br>18-34<br>N = 1,009,672	35-49<br>N = 711,077	50-59<br>N = 342,308	60-99<br>N = 366,870
**Number of negative SARS-CoV-2 tests (by end 2022)**					
0	81.8 (1,987,267)	83.1 (839,227)	81.5 (579,659)	79.8 (273,010)	80.5 (295,371)
1	13.1 (317,758)	12.4 (124,958)	13.2 (93,617)	14.2 (48,662)	13.8 (50,521)
2+	5.1 (124,902)	4.5 (45,487)	5.3 (37,801)	6.0 (20,636)	5.7 (20,978)
**COVID-19 Vaccination status[b] and boosters (at end 2022)**					
None	55.0 (1,336,826)	60.9 (614,851)	51.2 (363,908)	49.0 (167,646)	51.9 (190,421)
Incomplete BNT162b2	4.9 (118,559)	6.2 (62,962)	4.4 (31,589)	3.6 (12,315)	3.2 (11,693)
Complete Ad26.COV2S (no booster)	9.7 (236,725)	11.2 (112,933)	12.3 (87,290)	7.7 (26,221)	2.8 (10,281)
Complete Ad26.COV2S (and booster)	1.7 (41,196)	1.4 (14,200)	2.6 (18,363)	1.9 (6,450)	0.6 (2,183)
Complete BNT162b2 (no booster)	22.6 (548,914)	18.1 (183,198)	24.3 (172,761)	28.3 (96,908)	26.2 (96,047)
Complete BNT162b2 (and booster)	6.1 (147,707)	2.1 (21,528)	5.2 (37,166)	9.6 (32,768)	15.3 (56,245)
**Vital status (at end 2022)**					
Alive	95.4 (2,318,859)	98.7 (996,771)	97.2 (690,943)	94.0 (321,632)	84.4 (309,513)
Died: not COVID-19 related	4.1 (99,525)	1.2 (12,399)	2.6 (18,501)	5.3 (18,277)	13.7 (50,348)
Died: COVID-19 related	0.5 (11,543)	0.0 (502)	0.2 (1,633)	0.7 (2,399)	1.9 (7,009)
**SARS-CoV-2 diagnosis (by end 2022)**					
Not experienced	92.1 (2,237,396)	93.4 (943,413)	91.9 (653,256)	90.1 (308,580)	90.5 (332,147)
Experienced (2020-2022)	7.9 (192,531)	6.6 (66,259)	8.1 (57,821)	9.9 (33,728)	9.5 (34,723)
**COVID-19 related hospitalisation or death (by end 2022)**					
Not experienced	98.4 (2,391,586)	99.4 (1,003,799)	98.9 (702,936)	97.6 (334,057)	95.6 (350,794)
Experienced (2020-2022)	1.6 (38,341)	0.6 (5,873)	1.1 (8,141)	2.4 (8,251)	4.4 (16,076)
**COVID-19 related hospitalisation with severe disease or death (by end 2022)**					
Not experienced	99.3 (2,412,279)	99.9 (1,008,359)	99.6 (707,897)	98.8 (338,362)	97.5 (357,661)
Experienced (2020-2022)	0.7 (17,648)	0.1 (1,313)	0.4 (3,180)	1.2 (3,946)	2.5 (9,209)
**COVID-19 related death (by end 2022)**					
Not experienced	99.5 (2,418,384)	100.0 (1,009,170)	99.8 (709,444)	99.3 (339,909)	98.1 (359,861)
Experienced (2020-2022)	0.5 (11,543)	0.0 (502)	0.2 (1,633)	0.7 (2,399)	1.9 (7,009)

PHC: Primary healthcare

[a] Tuberculosis (at any time in past) includes those with an ongoing episode.

[b] Complete vaccination: ≥28 days since a first Ad26.COV2.S dose or ≥14 days since a second dose following a first BNT162b2 dose; incomplete BNT162b2: ≥21 days since a first BNT162b2 dose but not yet completely vaccinated (see methods).

S2.2. COVID-19 outcomes, SARS-CoV-2 testing and positivity, and COVID-19 vaccination over time

The figures reported in this section are similar to Figure 1 of the article. However, here we consider distinct (sub)cohorts within our study population, as defined by sex, age at the start of 2020, and HIV status at the start of 2020.

Note that the scales of the y-axes may vary by stratification variable.

Figure footnotes:

[a] Proportions are not shown for first and last months due to large uncertainties from few diagnoses/tests.

[b] A person is completely vaccinated ≥28 days since a first Ad26.COV2S dose or ≥14 days since a second dose following a first BNT162b2 dose; and is in the incomplete BNT162b2 state ≥21 days since a first BNT162b2 dose and while not yet completely vaccinated. 'No vaccine / transition' means the person has not received a dose or it is <28 days since a first Ad26.COV2S dose or <21 days since a first BNT162b2 dose. The time in the current vaccinated state (i.e. time since entering the state, which is reset 7 days following a booster dose) is also shown (< 6 versus ≥ 6 months). See methods of the article.

S2.2.1 Outcomes, testing and vaccination over time: Males

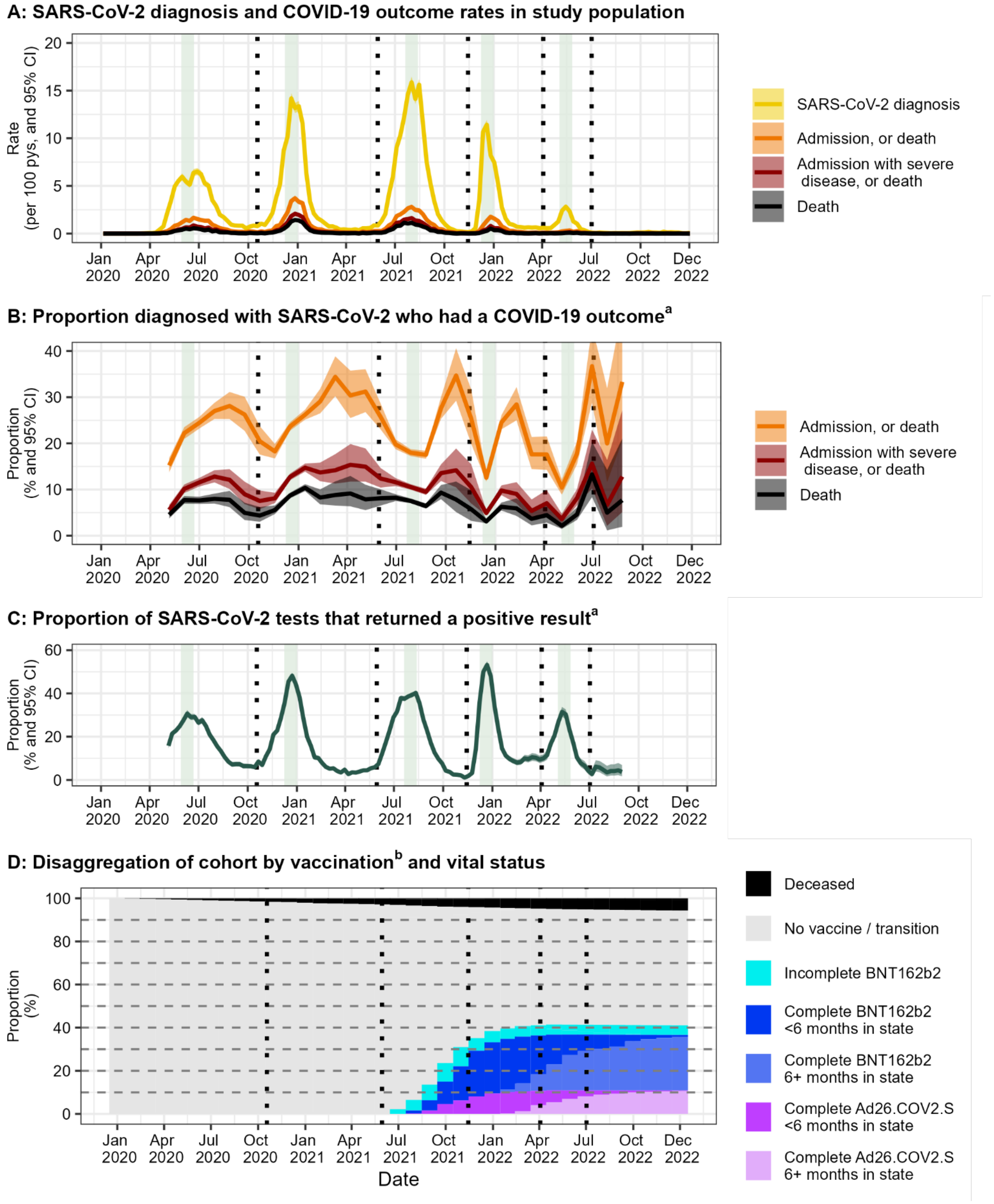

S2.2.2 Outcomes, testing and vaccination over time: Females

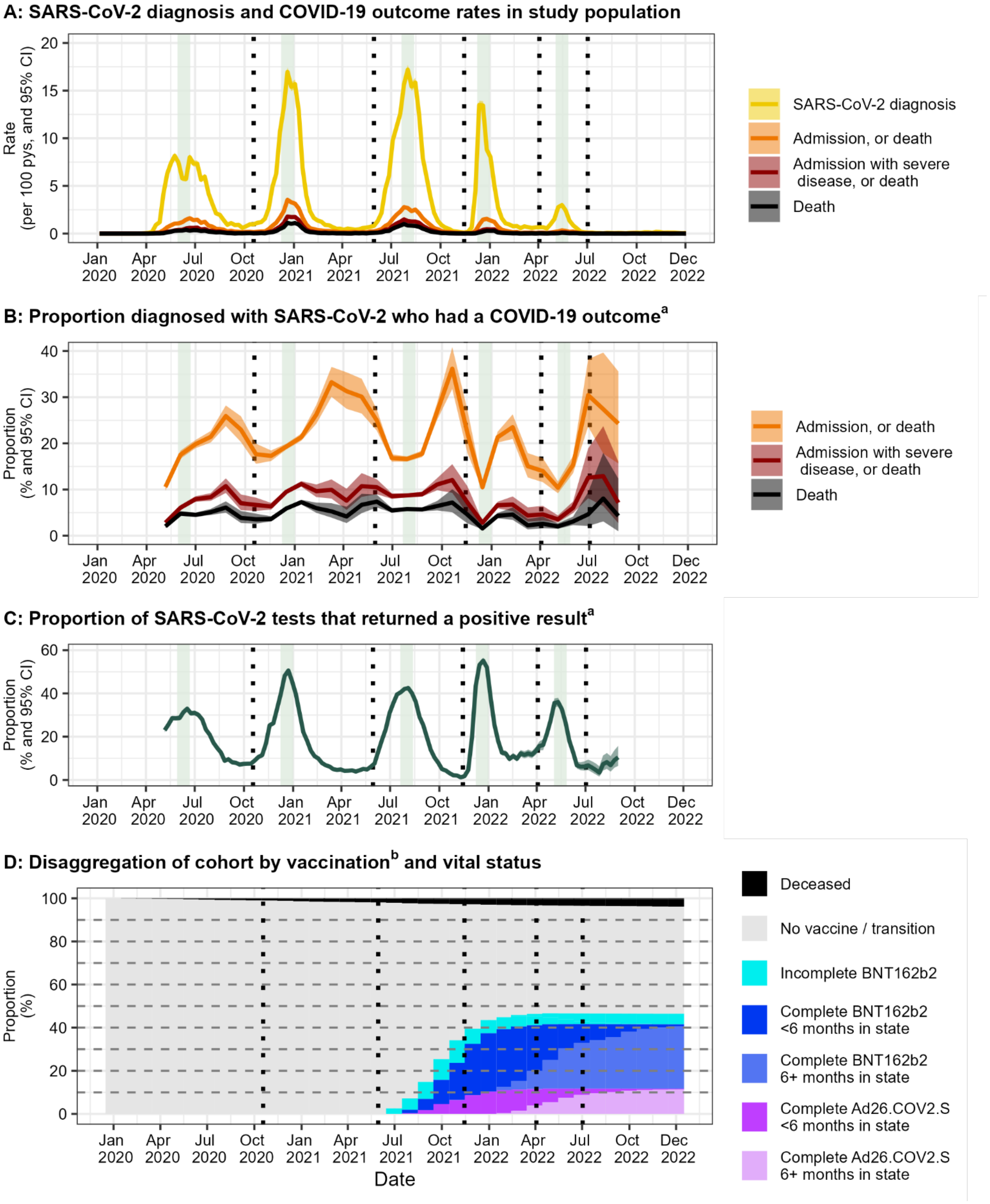

S2.2.3 Outcomes, testing and vaccination over time: Age 18-34 years

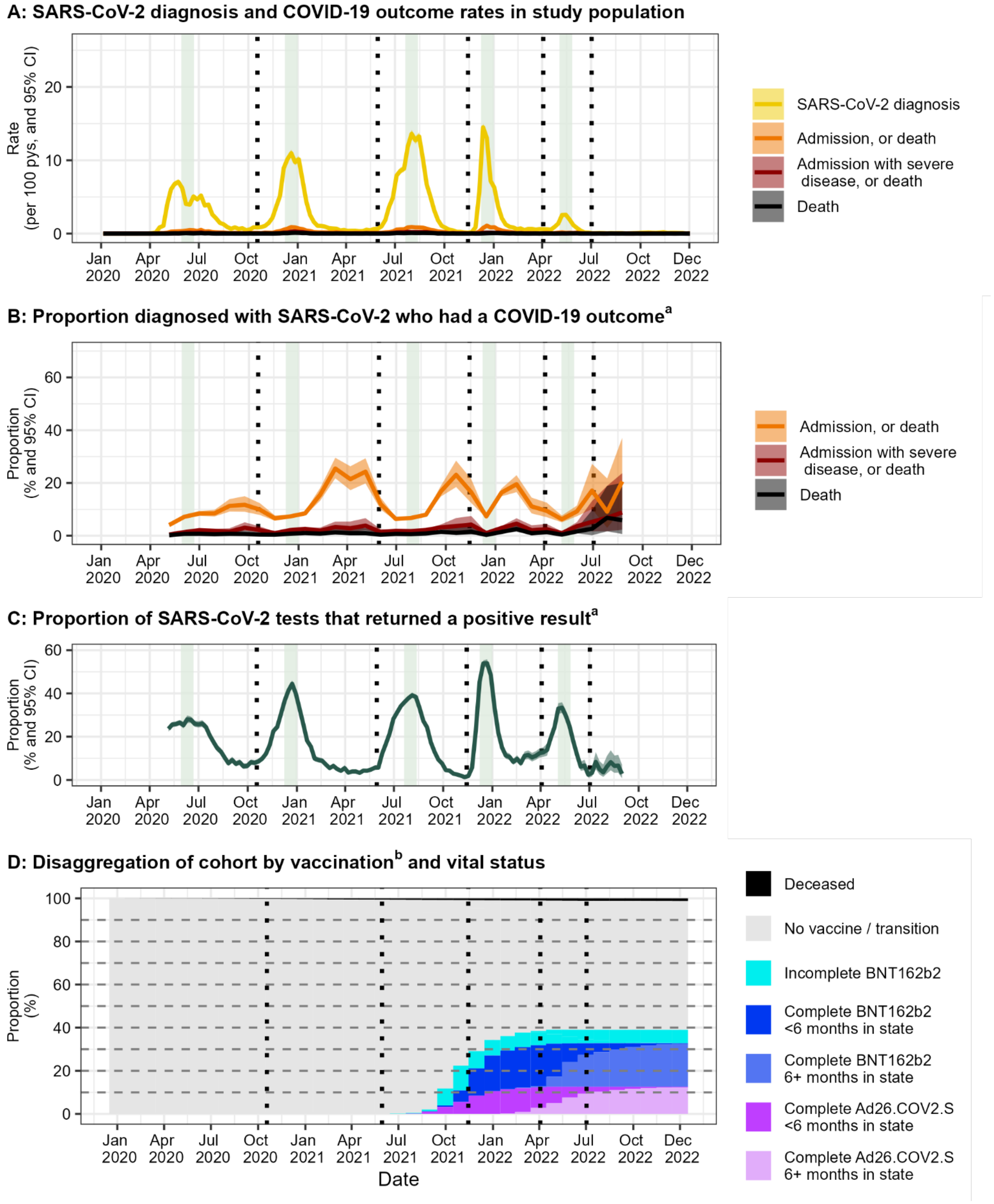

S2.2.4 Outcomes, testing and vaccination over time: Age 35-49 years

A: SARS-CoV-2 diagnosis and COVID-19 outcome rates in study population

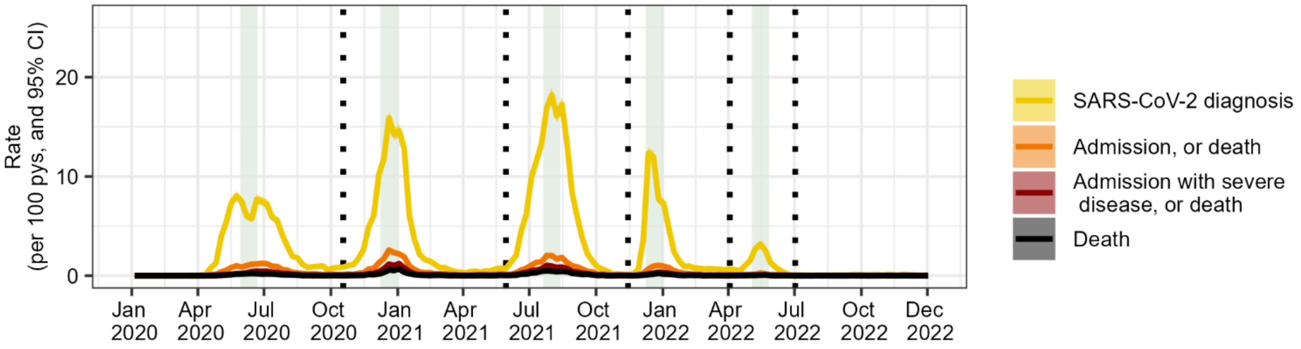

B: Proportion diagnosed with SARS-CoV-2 who had a COVID-19 outcome[a]

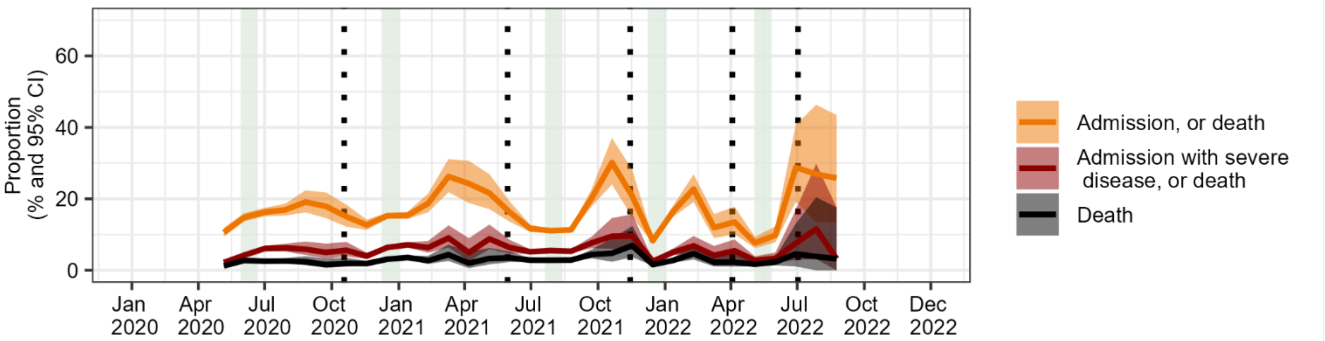

C: Proportion of SARS-CoV-2 tests that returned a positive result[a]

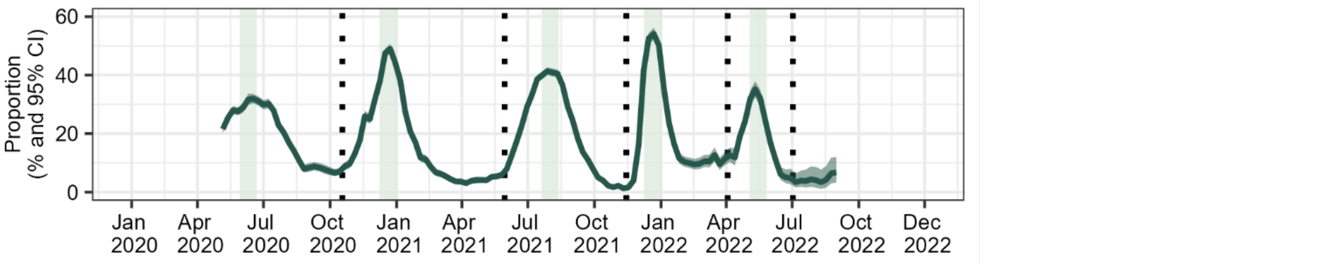

D: Disaggregation of cohort by vaccination[b] and vital status

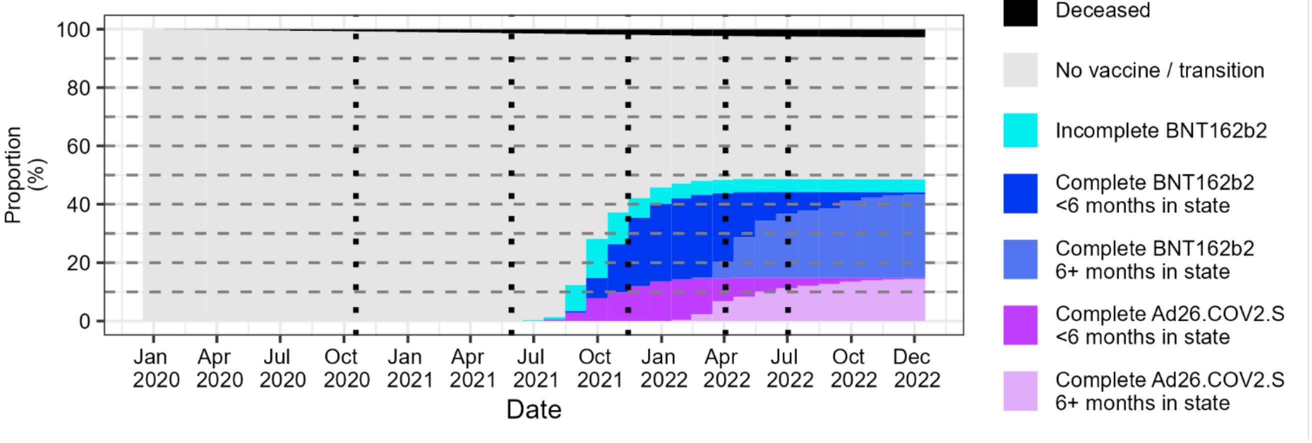

S2.2.5 Outcomes, testing and vaccination over time: Age 50-59 years

A: SARS-CoV-2 diagnosis and COVID-19 outcome rates in study population

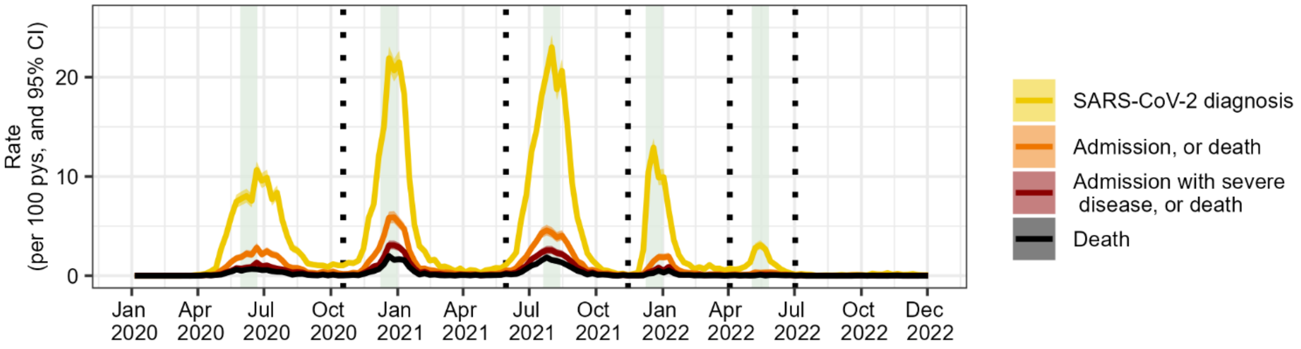

B: Proportion diagnosed with SARS-CoV-2 who had a COVID-19 outcome[a]

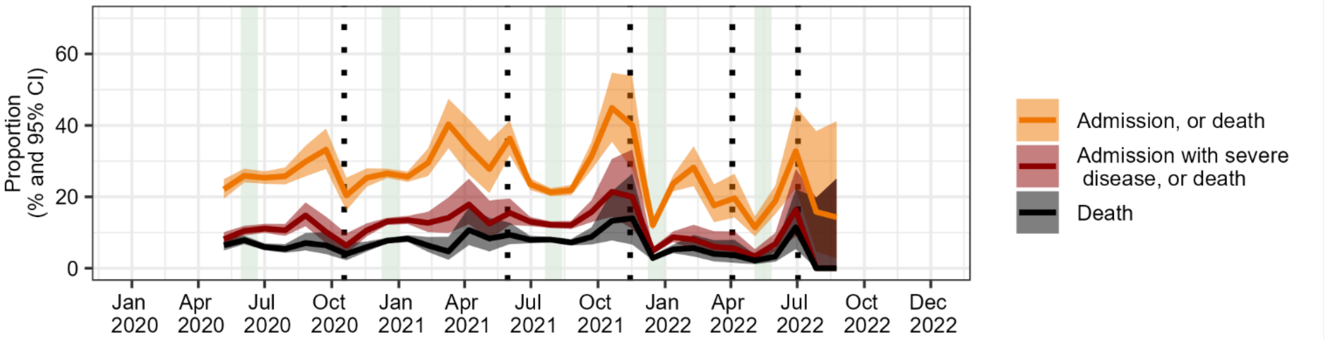

C: Proportion of SARS-CoV-2 tests that returned a positive result[a]

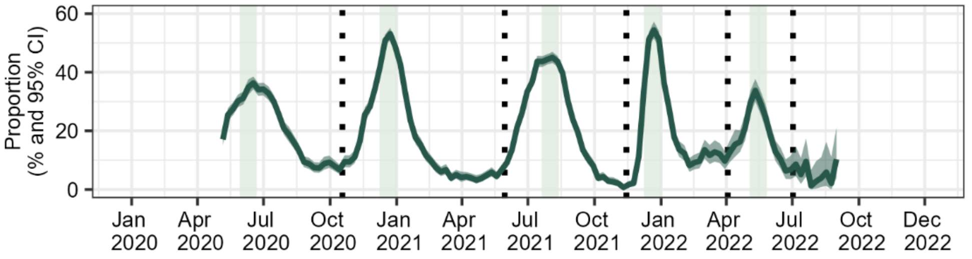

D: Disaggregation of cohort by vaccination[b] and vital status

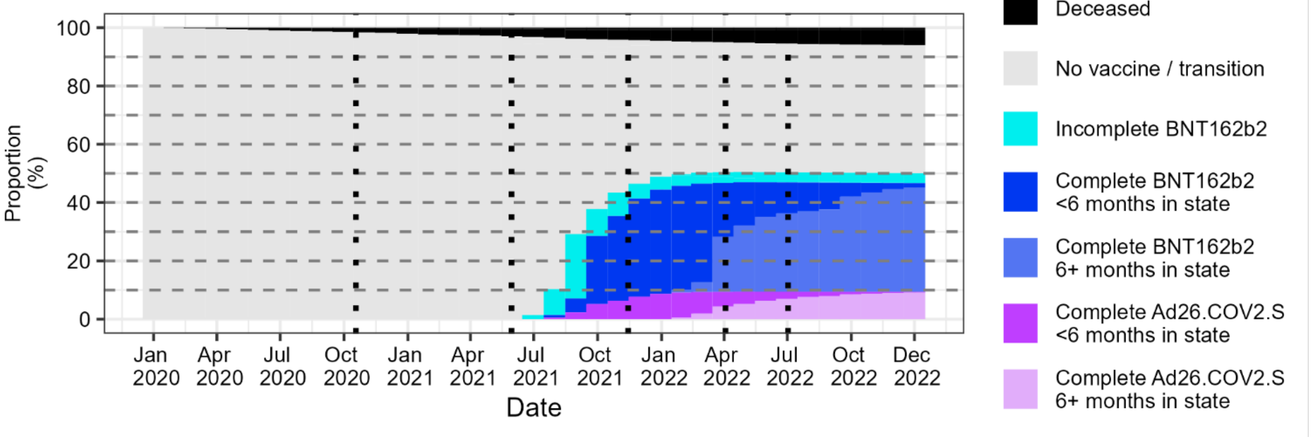

S2.2.6 Outcomes, testing and vaccination over time: Age 60+ years

A: SARS-CoV-2 diagnosis and COVID-19 outcome rates in study population

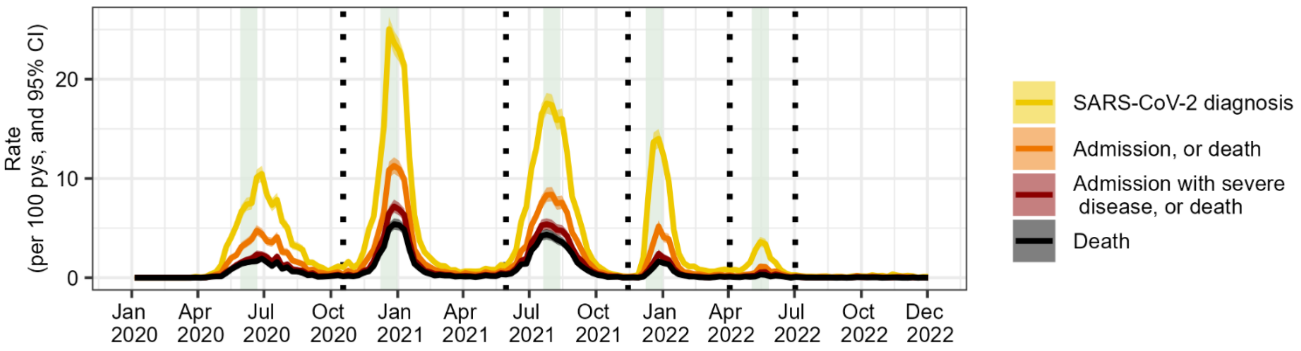

B: Proportion diagnosed with SARS-CoV-2 who had a COVID-19 outcome[a]

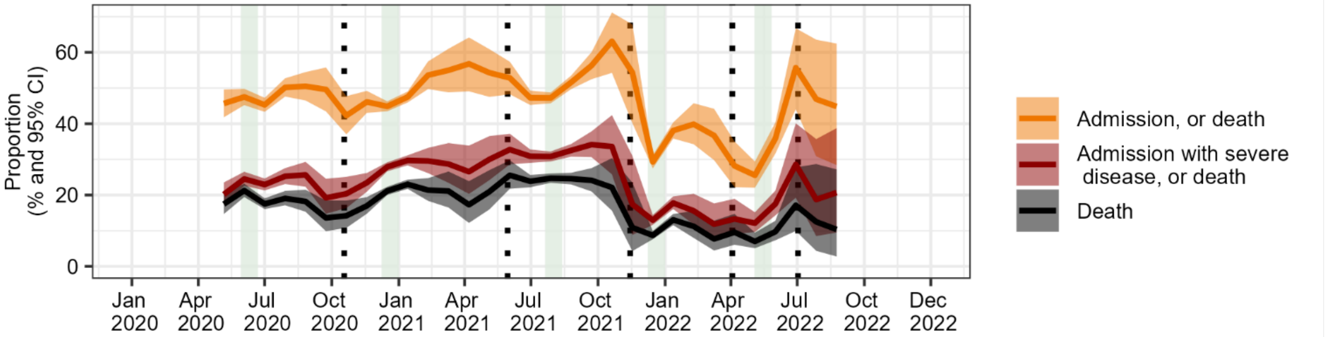

C: Proportion of SARS-CoV-2 tests that returned a positive result[a]

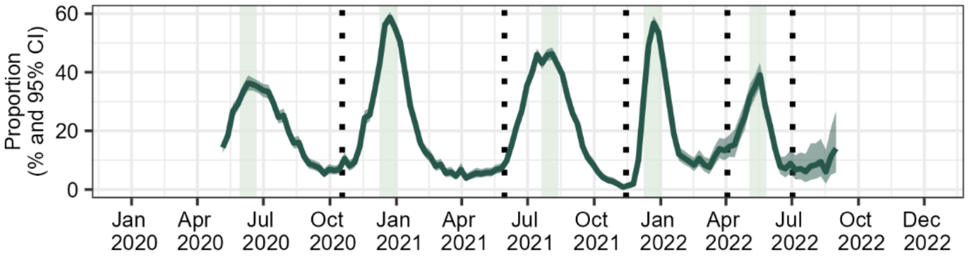

D: Disaggregation of cohort by vaccination[b] and vital status

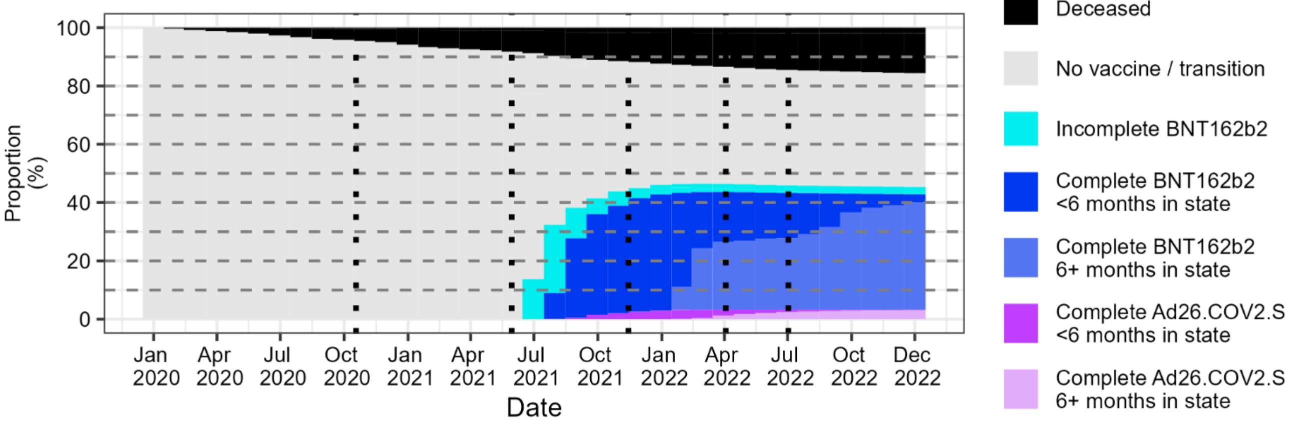

S2.2.7 Outcomes, testing and vaccination over time: Living with HIV at start 2020

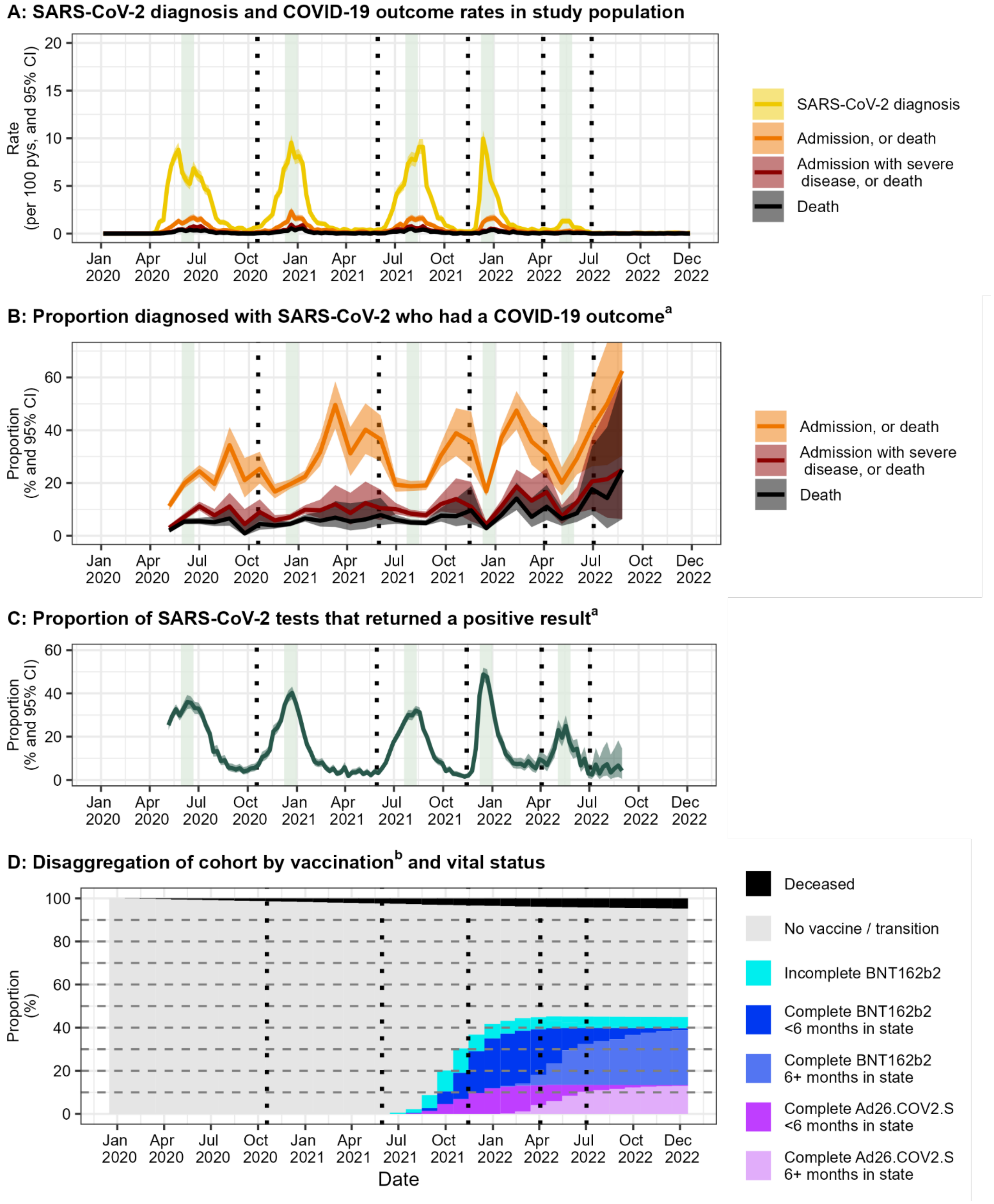

S2.2.8 Outcomes, testing and vaccination over time: Not known to be living with HIV at start 2020

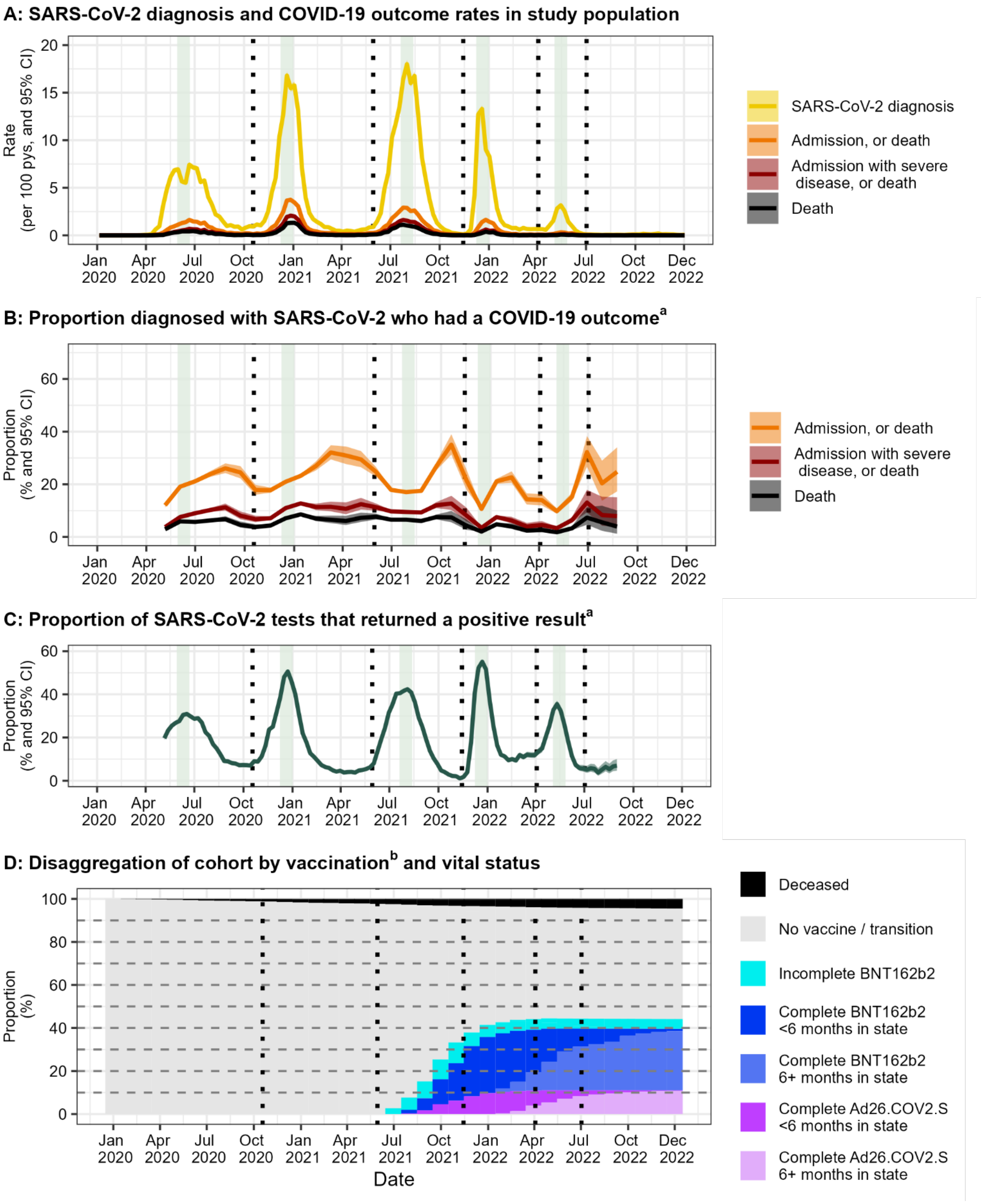

S2.3. Vaccine effectiveness for distinct subpopulations

The figures reported in this section are similar to Figure 3 in the article.

However, here we consider distinct (sub)cohorts within our study population, as defined by sex, age at the start of 2020 and HIV status at the start of 2020. We also report vaccine effectiveness when excluding people who received heterologous doses, from the time they received their first dose.

S2.3.1 Vaccine effectiveness: Males

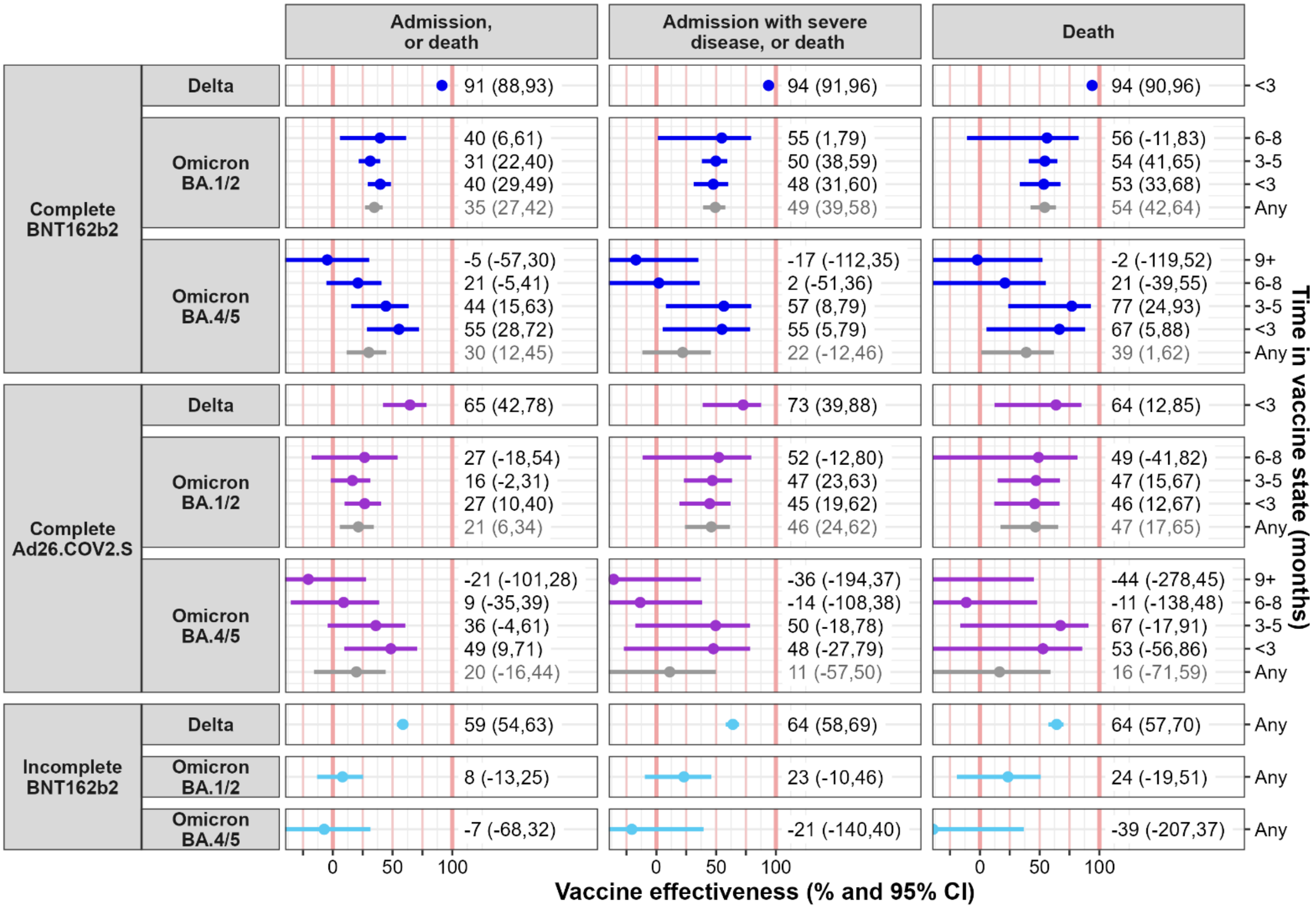

S2.3.2 Vaccine effectiveness: Females

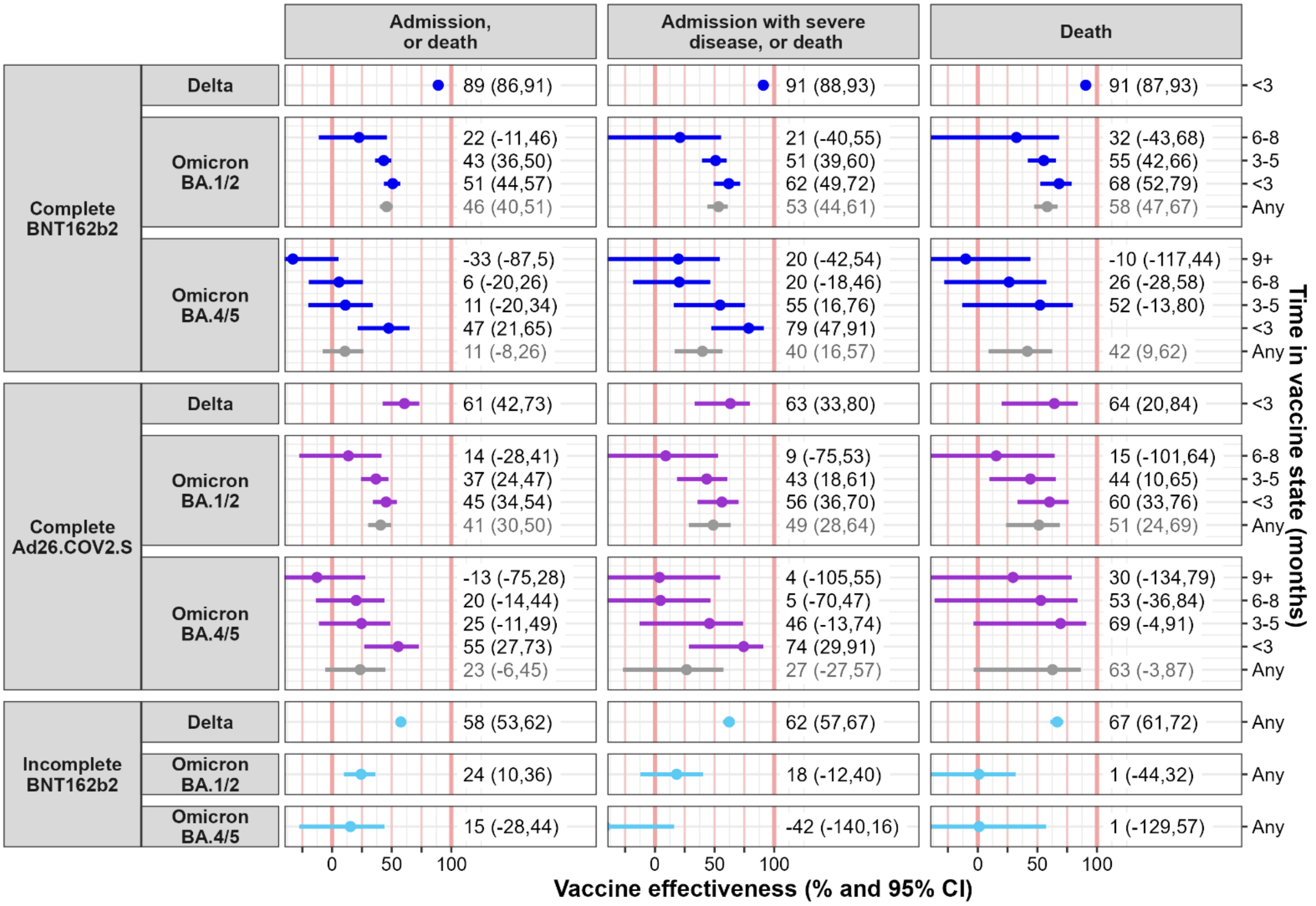

 Supplementary Material 2: Analysis Outputs
See Kassanjee et al. COVID-19 vaccine uptake and effectiveness by time since vaccination in the Western Cape province, South Africa: An observational cohort study during 2020-2022.

S2.3.3 Vaccine effectiveness: Age 18-49 years

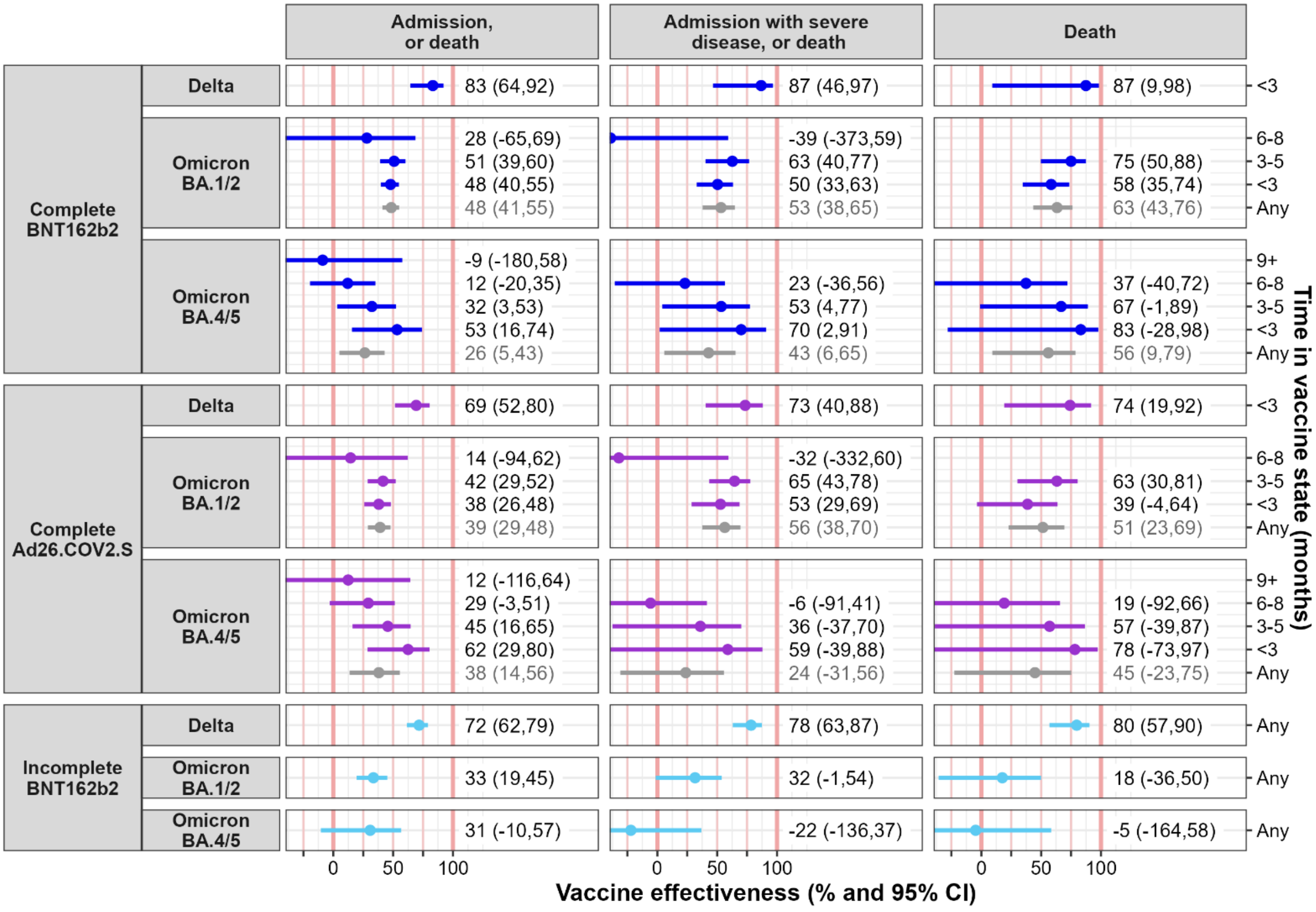

S2.3.4 Vaccine effectiveness: Age 50+ years

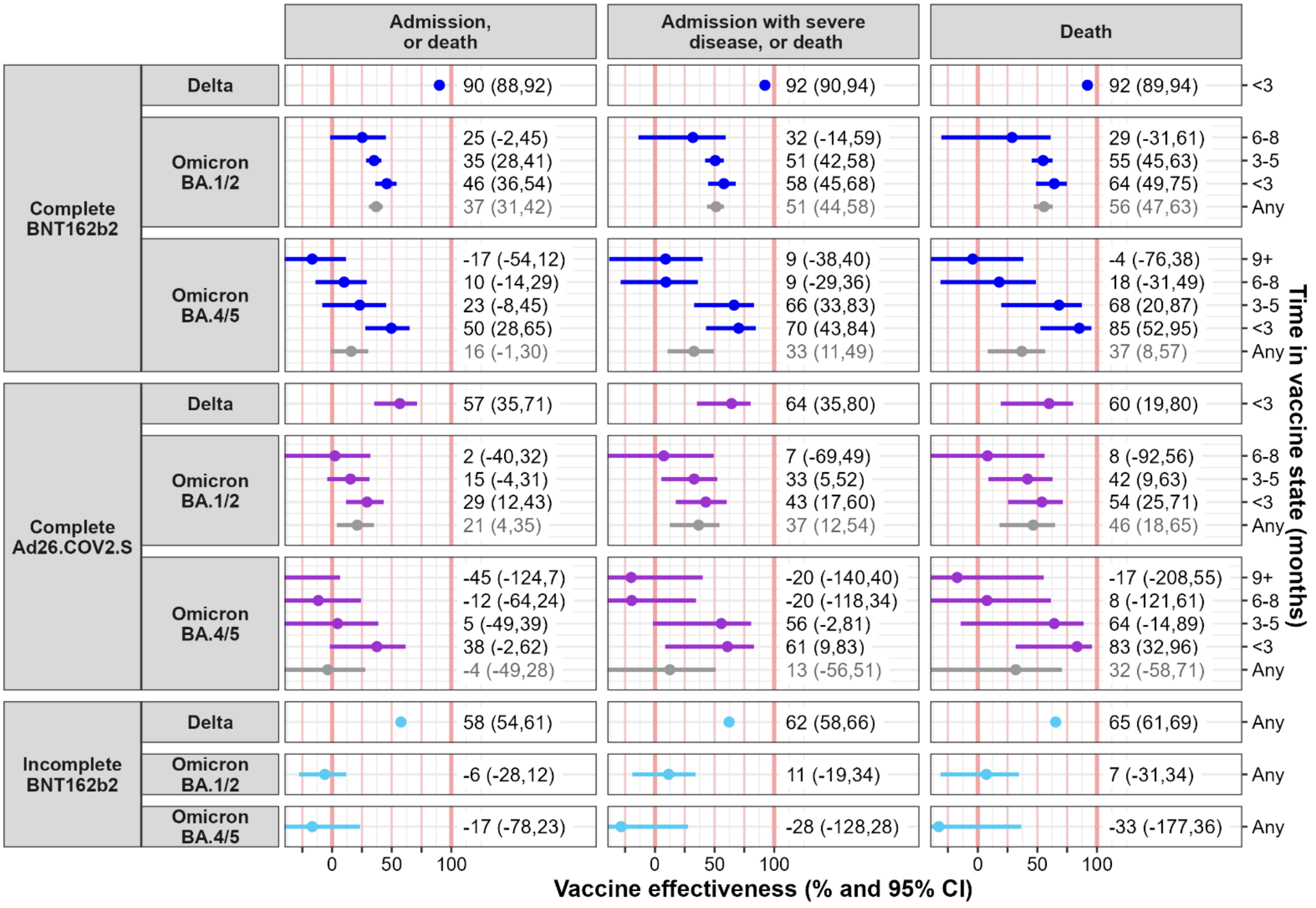

S2.3.5 Vaccine effectiveness: Known to be living with HIV at start 2020

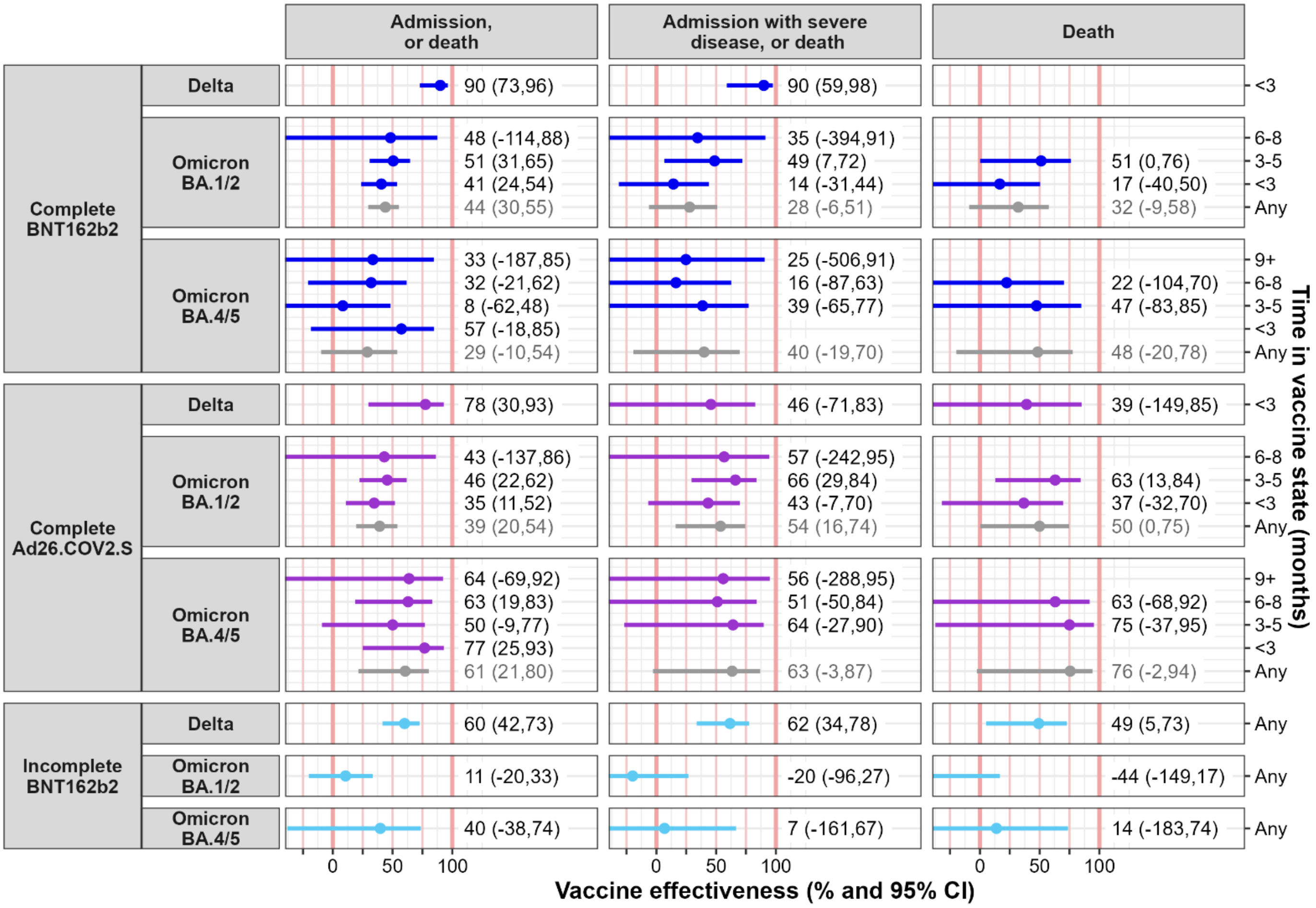

S2.3.6 Vaccine effectiveness: Not known to be living with HIV at start 2020

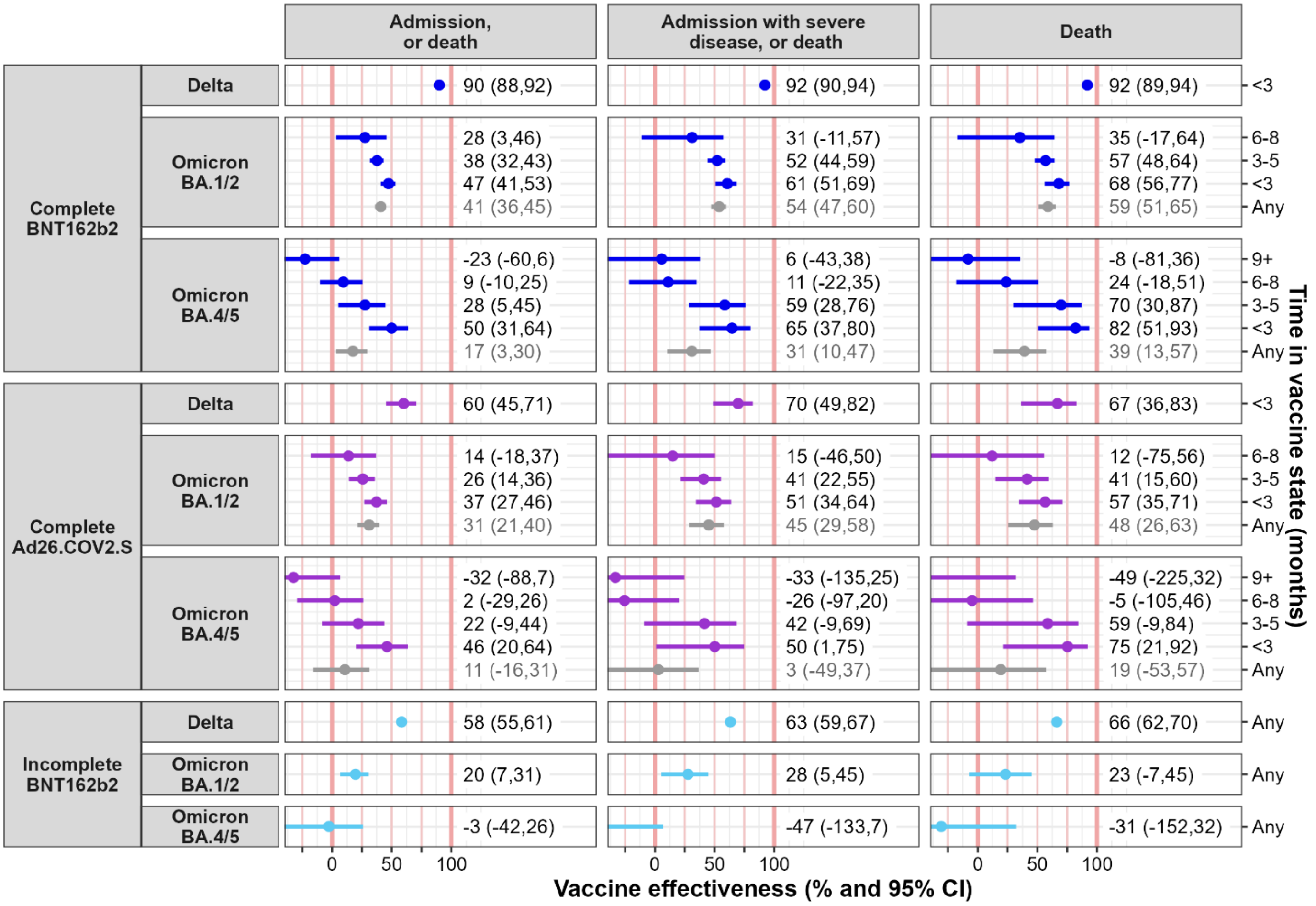

S2.3.7 Vaccine effectiveness: Excluding people who received heterologous vaccines

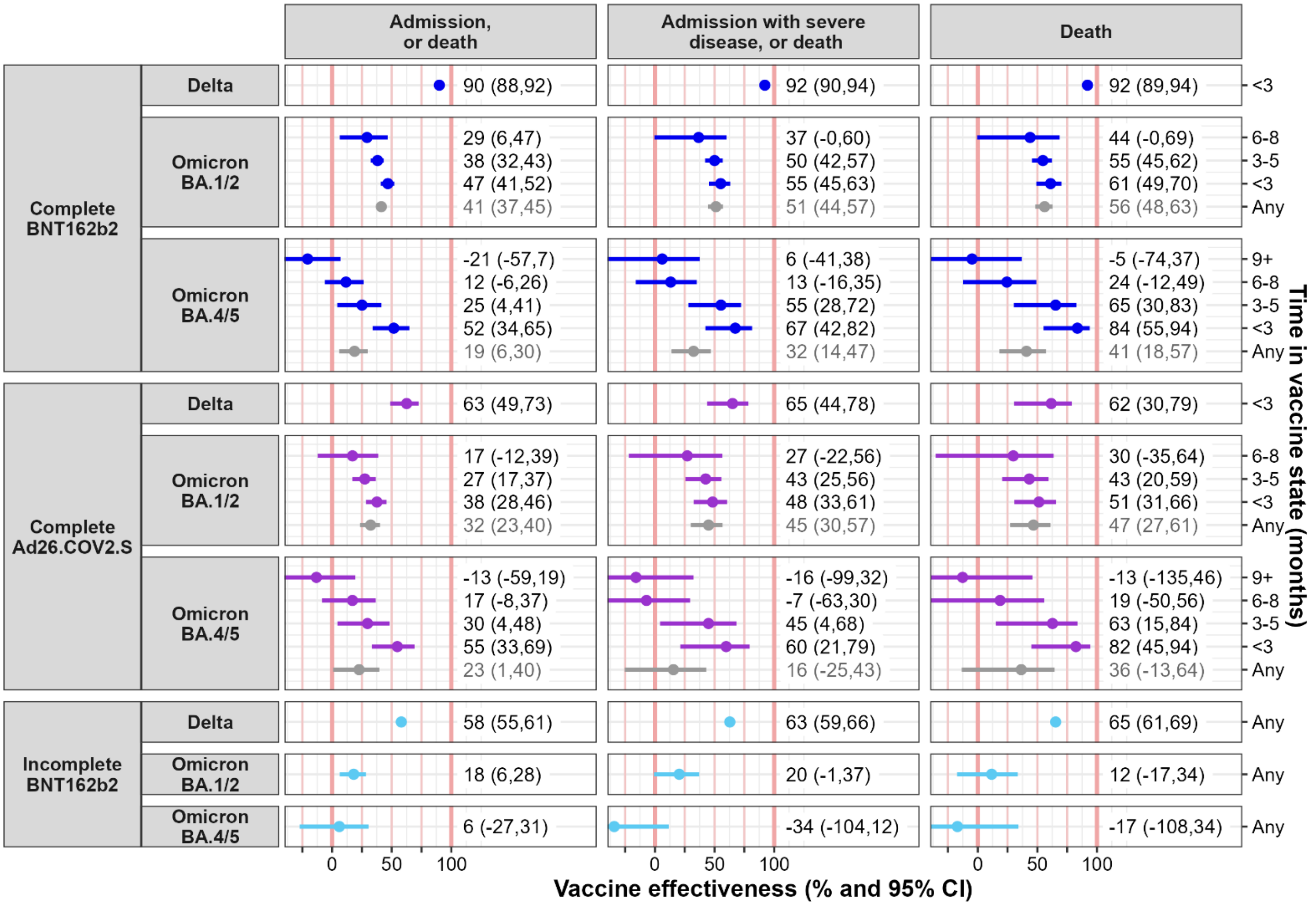

People who received heterologous doses were excluded from the time of receiving their first dose.

Among those people alive at start 2020 who completed their BNT162b2 primary vaccination series by end 2022, 28 of 696,621 (0.004%) persons received a heterologous second dose. Considering all 1,093,101 people who received at least one dose by end 2022 (incomplete or complete primary vaccination series, with or without boosters), 1.43% received heterologous doses (28 people as part of the primary series, and the rest during boosting).

S2.4. All multivariable cox model hazard ratios

For completeness of reporting, the fitted hazard ratios for all model terms (vaccination terms and covariates) in the multivariable cox model used to estimate vaccine effectiveness are reported below. All hazard ratios reported in the section are from the same multivariable model, which adjust for all covariates.

For each set of model terms, the hazard ratio (x-axis) is shown for the different model terms (figure rows) and COVID-19 outcomes (figure columns), for the three wave periods that occurred after vaccine rollout (legend). The hazard ratio is the rate of the outcome in a category of persons, relative to a reference category (as indicated in the figure row label), holding all other model terms fixed. Hazard ratios greater than 1 represent higher outcome rates; and less than 1 lower outcome rates.

The focus of the article is on reporting the vaccine effectiveness estimates. The direct interpretation of the hazard ratios for the covariates presented below are complex and outside of the focus of this work: for example, a higher rate out of an outcome in a category of persons may results from more exposure to the virus (e.g. limited opportunity to socially distance), a greater probability of COVID-19 disease (or more severe disease) given that the person has acquired the SARS-CoV-2 virus, greater SARS-CoV-2 testing, or an association of COVID-19 outcomes with a variable for which the covariate is only a proxy or associated variable.

S2.4.1 Hazard ratios: Demographic characteristics

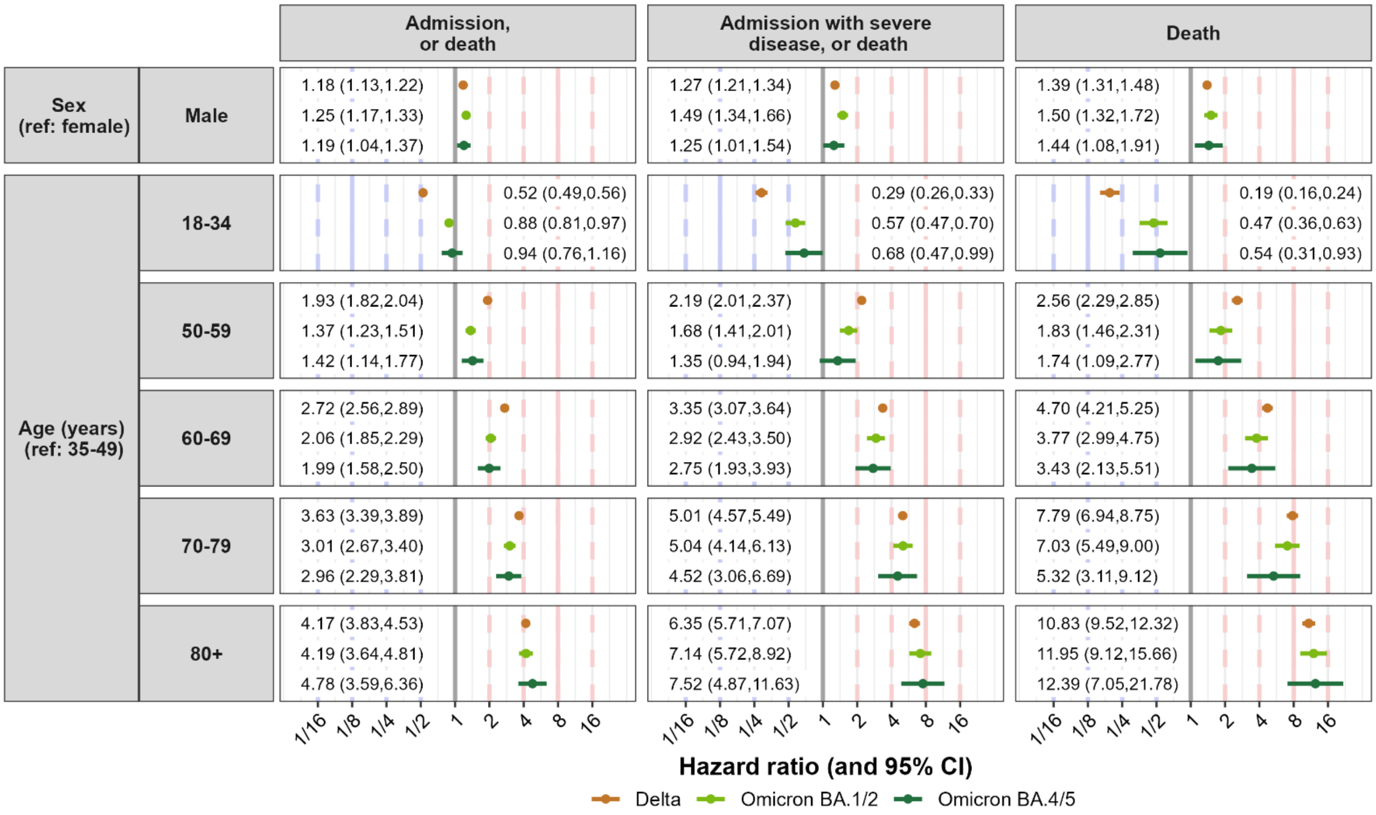

S2.4.2 Hazard ratios: Non-communicable conditions

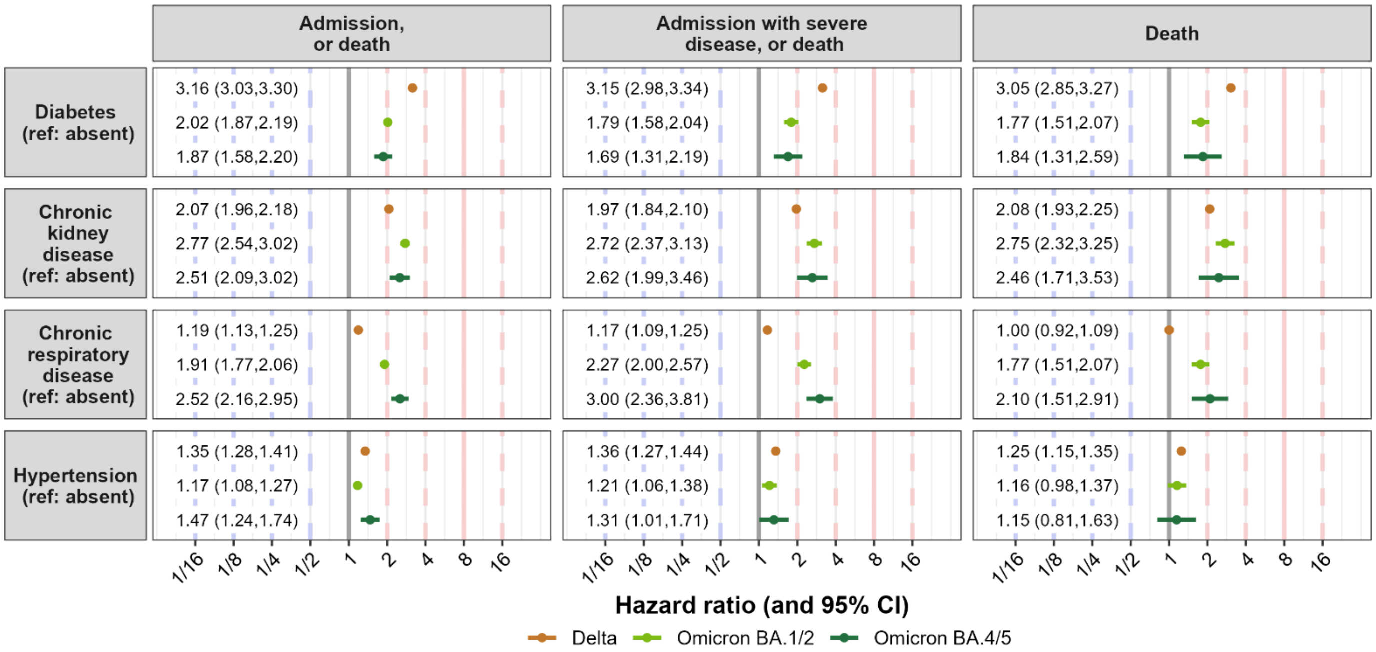

S2.4.3 Hazard ratios: Infectious conditions

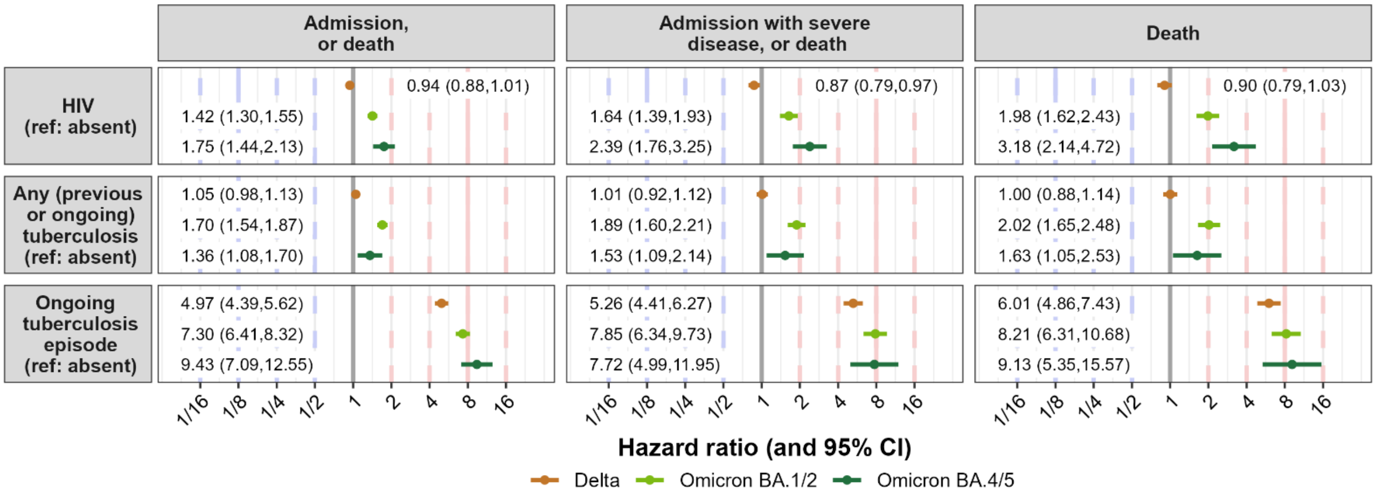

'Any' tuberculosis includes those with a previous or ongoing episode – i.e. the effect of an 'ongoing tuberculosis episode' is the *additional* effect of an episode being ongoing rather than only at some point in the past.

S2.4.4 Hazard ratios: Other conditions

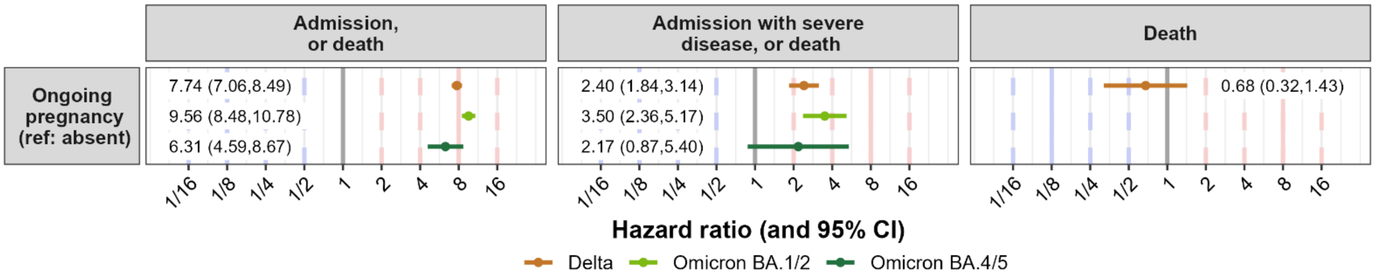

S2.4.5 Hazard ratios: Prior SARS-CoV-2 diagnosis

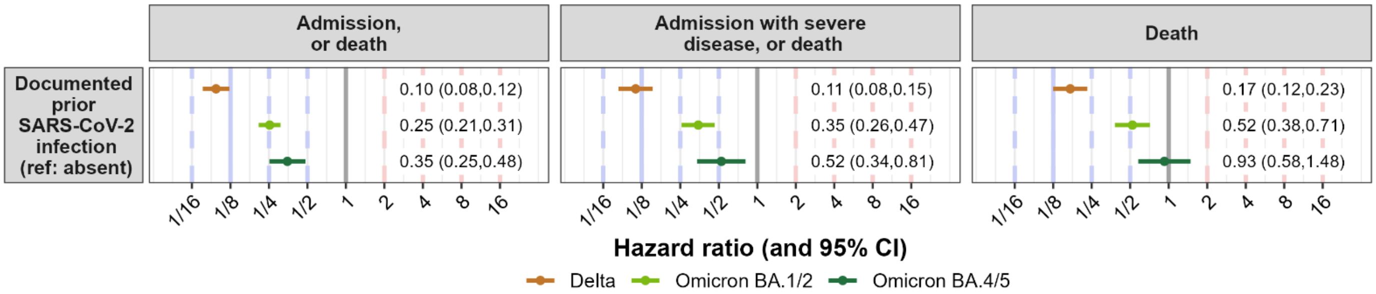

S2.4.6 Hazard ratios: Location

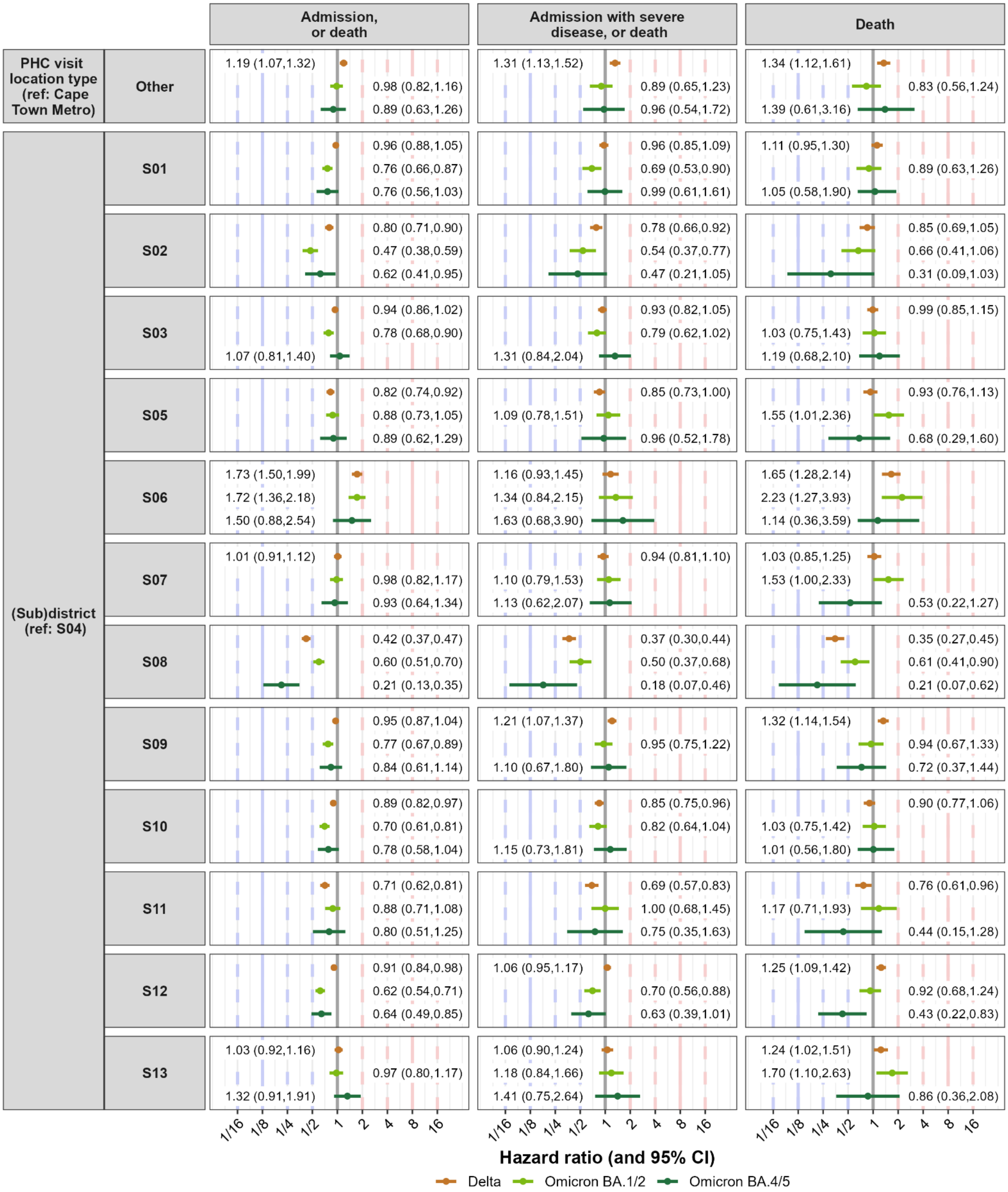

For PHC (primary healthcare) visit location type, 'Other' includes non-Metro and no recorded visit.

S2.4.7 Hazard ratios: Healthcare utilisation and testing

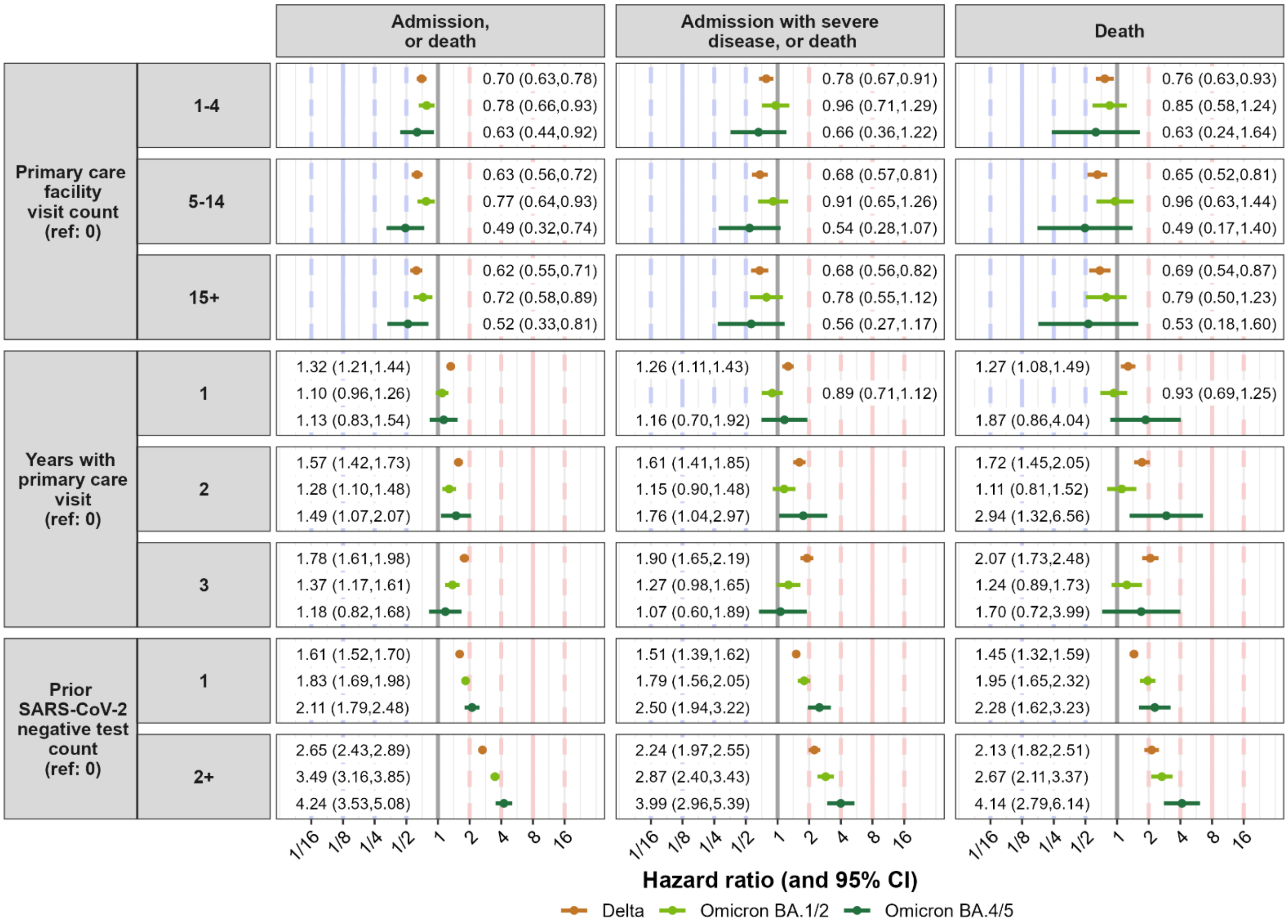

Supplementary Material 2: Analysis Outputs

See Kassanjee et al. COVID-19 vaccine uptake and effectiveness by time since vaccination in the Western Cape province, South Africa: An observational cohort study during 2020-2022.

S2.4.8 Hazard ratios: Vaccine terms

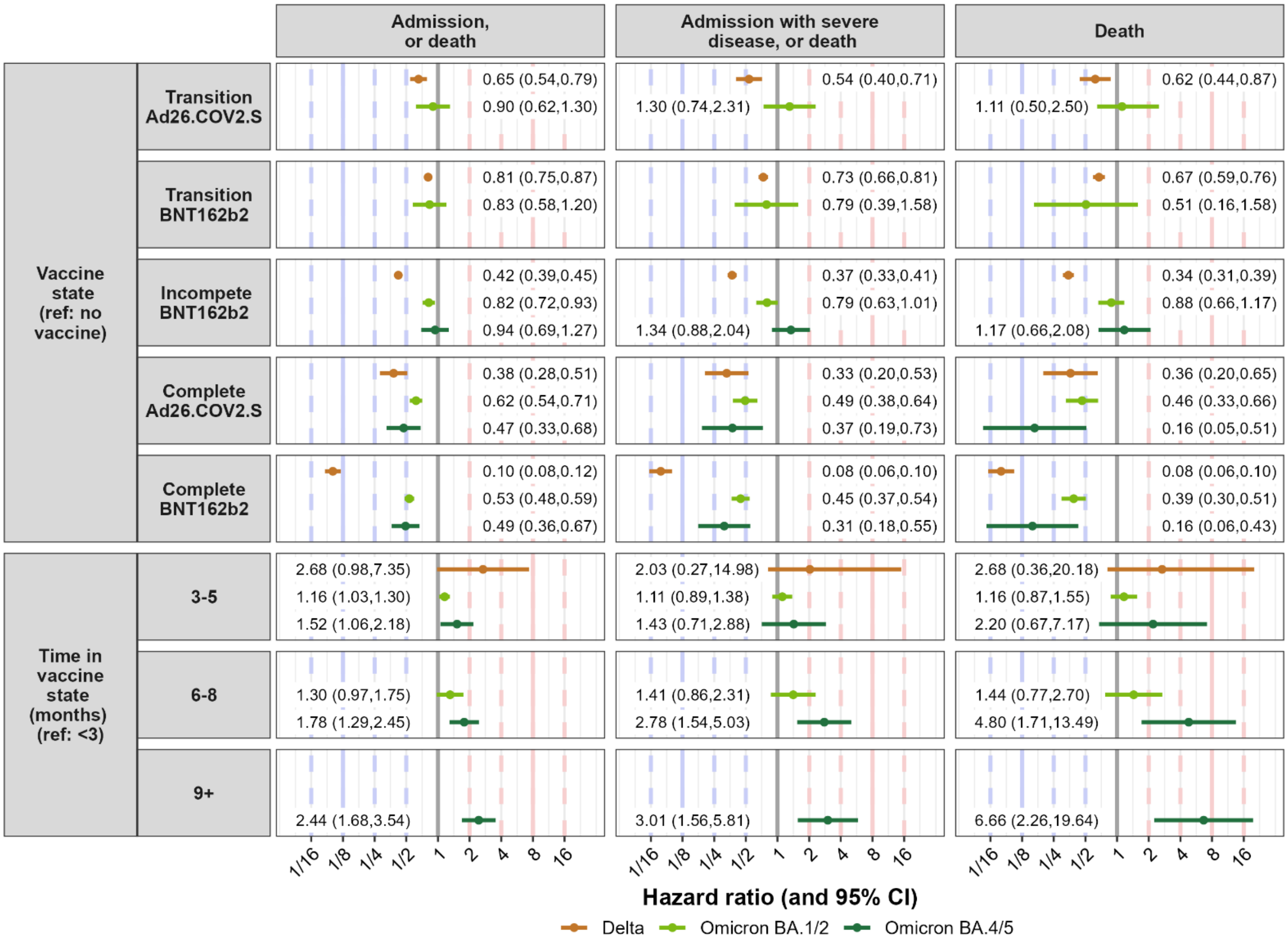

S2.5. Vaccine effectiveness for rolling windows of calendar time

To understand how the associations between vaccine status and COVID-19 outcomes change over calendar time, the multivariable cox model was fitted to rolling windows of calendar time – each of duration 6 weeks, and each window starting 3 weeks after the previous window began. Due to limited precision when using data from short windows of time, vaccine state was not further distinguished by time in the vaccine state (time in vaccine state was included in the primary model reported in the article).

Changes in associations of vaccination with severe COVID-19 over calendar time are likely due to many factors, such as variations in time since most recent vaccine dose, testing coverage and match of vaccine and dominant infection (sub)lineage.

The figure below reports vaccine effectiveness estimates (x-axis) for each vaccinated state (figure rows) and COVID-19 outcome (figure columns), over time (y-axis, midpoint of window). The horizontal shaded areas (orange, light green, dark green) correspond to the dominant (sub)lineage in different periods (i.e., the distinct analysis wave periods used in the primary analysis). The grey curved line represents a locally weighted smoothing of the trend, to informally guide the eye. Vaccine effectiveness is 1 minus the ratio of rates of outcomes in vaccinated persons versus unvaccinated persons, expressed as a percentage: i.e. 100% is perfect 'protection' and 0% is no 'protection', shown by vertical red lines.

A person is completely vaccinated ≥28 days since a first Ad26.COV2S dose or ≥14 days since a second dose following a first BNT162b2 dose; and is in the incomplete BNT162b2 state ≥21 days since a first BNT162b2 dose and while not yet completely vaccinated.

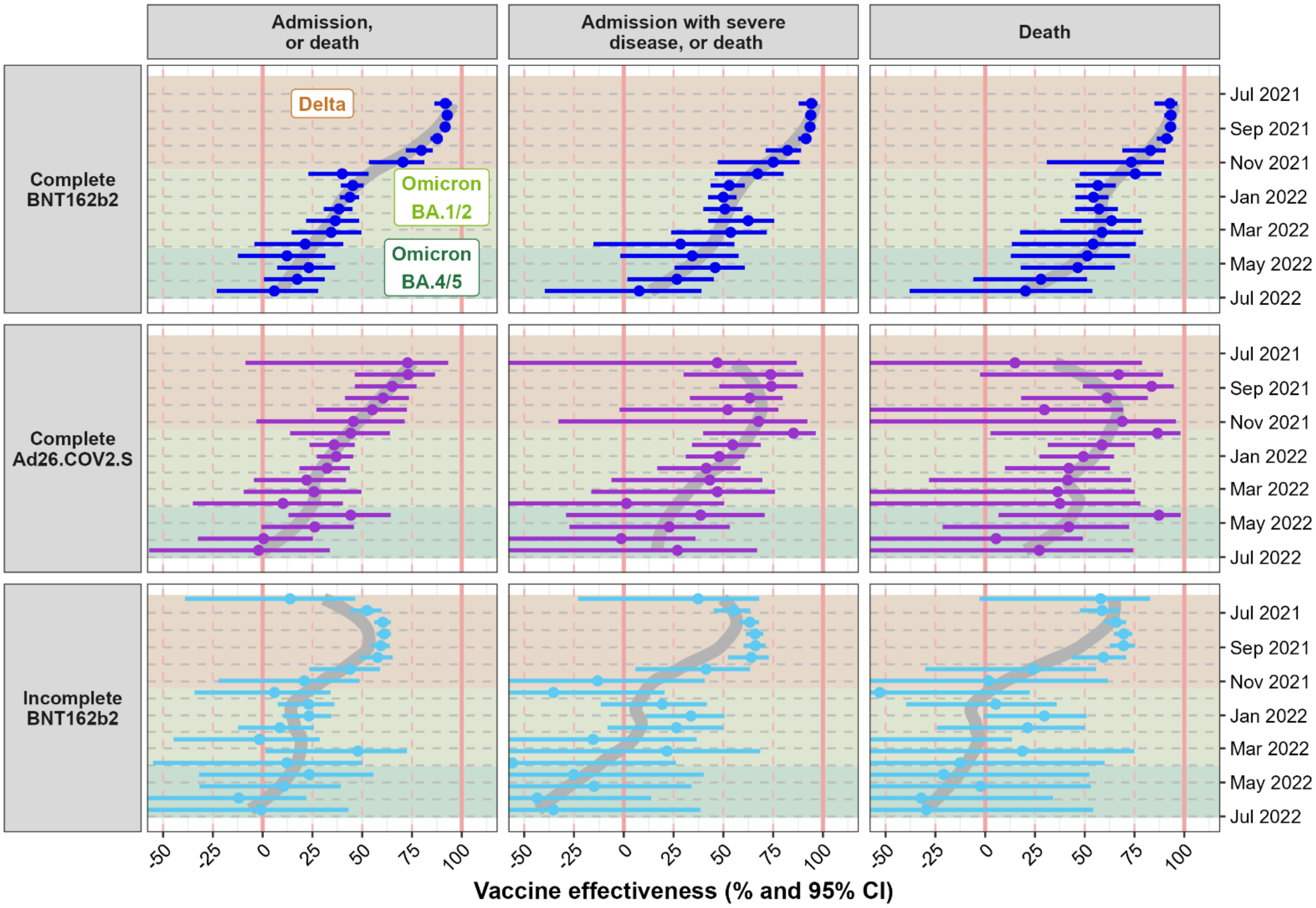

Supplement: Supplementary file 1 [file vaccines-12-00628-s001.zip › SupplementaryMaterial File S2_AnalysisOutputs.pdf]
